# A Modified Multi-Strategy Dhole Optimization Algorithm and Its Engineering Applications

**DOI:** 10.3390/biomimetics11060436

**Published:** 2026-06-18

**Authors:** Jingya Zhang, Yu Liu, Chaochuan Jia, Maosheng Fu, Yaqi Yang, Jiahui Liu, Yujie Cheng

**Affiliations:** 1 School of Electronic Information and Artificial Intelligence, West Anhui University, Lu’an 237012, China; 2 Anhui Province Intelligent Hydraulic Machinery Joint Construction Subject Key Laboratory, Lu’an 237012, China

**Keywords:** Modified Dhole Optimization Algorithm, multi-strategy fusion, engineering optimization, CEC benchmark functions, moisture content prediction

## Abstract

To address the inherent limitations of the Dhole Optimization Algorithm (DOA)—limited exploration range, insufficient population diversity, and slow convergence—this paper proposes a Modified Dhole Optimization Algorithm (MDOA) integrating a Beta distribution-based opposition learning strategy, a DE/rand-to-best/1 differential mutation mechanism, and nonlinear parameter control. MDOA is evaluated on 41 CEC2017 and CEC2022 benchmark functions, outperforming 11 state-of-the-art algorithms in convergence speed, accuracy, and robustness. It is then applied to five engineering optimization problems: compression spring design, speed reducer weight minimization, rolling bearing optimization, tubular column design, and moisture content prediction of Dendrobium huoshanense using near-infrared spectroscopy with a BP neural network. The MDOA-BP model reduces MAE, RMSE, MSE, and MAPE by 27.5%, 27.8%, 47.6%, and 31.0%, respectively, while increasing $R^{2}$ from 0.8339 to 0.9130, achieving the best results among all comparison models. These results demonstrate that MDOA is a highly effective and robust optimizer for complex constrained engineering and high-dimensional optimization tasks.

## 1. Introduction

In recent years, improving existing metaheuristic algorithms has become a more efficient and widely recognized approach than proposing entirely new ones [1,2,3,4,5,6]. Introducing opposition-based learning mechanisms, designing adaptive parameter adjustment strategies, and integrating differential mutation operators have been shown to effectively enhance population diversity, convergence speed, and the ability to escape local optima. By systematically integrating multiple strategies, enhanced algorithms can retain their original advantages while addressing deficiencies in convergence accuracy and global optimization capability.

In recent years, many novel metaheuristic algorithms have been proposed and validated on complex optimization problems. These can be roughly classified into categories inspired by predation behavior, swarm collaboration, and mathematical or physical principles.

Predation-inspired algorithms include the Black-winged Kite Algorithm (BKA) [7], Harris Hawks Optimization (HHO) [8], the Parrot Optimizer (PO) [9], the Sparrow Search Algorithm (SSA) [10], the Secretary Bird Optimization Algorithm (SBOA) [11], and Birds of Prey-Based Optimization (BPBO) [12]. Group collaboration-inspired algorithms include Beluga Whale Optimization (BWO) [13], the Coati Optimization Algorithm (COA) [14], and the Dung Beetle Optimizer (DBO) [15]. Mathematics- and physics-inspired algorithms include the Golden Sine Algorithm (GoldSA) [16], the Tornado Optimizer with Coriolis Force (TOC) [17], and Leaf in Wind Optimization (LiWO) [18]. Additionally, Particle Swarm Optimization (PSO) [19] is a classical swarm intelligence algorithm based on information sharing.

To address these issues, researchers have proposed various improved algorithms. Liu et al. enhanced the Subtraction Average-based Optimizer with nonlinear control parameters and an adaptive mutation strategy to improve convergence accuracy [20]. Zhu et al. developed an Improved Snake Optimizer (ISO) using a multi-strategy chaotic system, an anti-predator strategy, and bidirectional population evolution dynamics [21]. Qi et al. introduced a stagnation-triggered diversification mechanism and an adaptive weak guidance mechanism to improve the Black-winged Kite Algorithm [22]. Zhang et al. proposed a Multi-strategy Improved Dung Beetle Optimizer (MIDBO) integrating piecewise mapping, quasi-opposition-based learning, a differential evolution mechanism, an adaptive golden sine strategy, and the inverse Cauchy cumulative distribution function, demonstrating excellent convergence accuracy and stability [23]. Li et al. developed a Multi-Strategy Improved Sparrow Search Algorithm (MSISSA) incorporating an adaptive weight operator, the spiral flight mechanism of the Moth-Flame Optimization Algorithm, and the Levy flight mechanism, showing significant advantages in three-dimensional wireless sensor node deployment [24]. Beşkirli and Dağ applied the original TSA to photovoltaic model parameter extraction, indicating the applicability of TSA-based search mechanisms in engineering parameter optimization problems [25]. They also proposed I-CPA by adding a teaching factor strategy to CPA, improving its local search capability and stability on CEC2017 functions and photovoltaic parameter identification problems [26]. Subsequently, Beşkirli et al. developed MS-TSA by incorporating multiple enhancement strategies into TSA for photovoltaic model parameter estimation [27]. Gao et al. proposed an Improved Coati Optimization Algorithm (ICOA) using a refraction opposite learning strategy, an enhanced Levy flight mechanism, and a variable spiral search mechanism, achieving a 16.8% improvement in signal-to-noise ratio and a 12.5% reduction in training time in milling chatter detection [28]. Saad et al. extended the Parrot Optimization Algorithm to a multi-objective version (Multi-objective Parrot Optimizer, MOPO) by introducing an external archive and a non-dominated sorting mechanism, demonstrating excellent performance on CEC2020 multi-objective benchmark tests and helical spring design [29].

Researchers have also improved algorithm performance through parameter adaptive adjustment and opposition-based learning. The Enhanced Gaining-Sharing Knowledge-based algorithm (eGSK) addresses premature convergence by adjusting selection criteria, modifying parameter settings, and escaping local optima [30]. Mohapatra et al. proposed a fast random opposition-based learning golden jackal optimization algorithm (FROBL-GJO), introducing randomness and a fast convergence strategy into opposition-based learning to enhance search efficiency and solution accuracy [31]. Zhao et al. introduced quadratic opposition-based learning and a random differential mutation mechanism into the predator–prey optimization algorithm, proposing a dual-quaternion hand–eye calibration method (ODHPO). This mechanism enhances population ergodicity through twice opposition-based learning and improves local exploitation via random differential mutation, effectively addressing the nonlinear optimization problem in hand–eye calibration [32].

The Dhole Optimization Algorithm (DOA) is a recent swarm intelligence method inspired by the tracking, encircling, and attacking behaviors of dhole packs [33]. DOA uses acoustic communication signals to adjust its search: weak signals trigger dispersed global search, while approaching the optimum enables fine-grained local exploitation. However, DOA has three shortcomings: (1) a simple vector updating mechanism limits the search scope in high-dimensional or strongly coupled problems and causes rapid diversity loss; (2) sine–cosine perturbations provide limited local search precision and slow later-stage convergence; (3) linear decay of control parameters fails to balance early exploration and later exploitation.

To address these issues, this paper proposes a Modified Dhole Optimization Algorithm (MDOA). It introduces a Beta distribution-based opposition learning strategy to expand the search range and enhance population diversity by adaptively generating concave or convex Beta-distributed oppositional solutions. It also integrates the DE/rand-to-best/1 differential mutation mechanism to improve local exploitation efficiency and accelerate convergence. Additionally, the original linearly decaying shrinkage coefficient is replaced with a nonlinear form, enabling a more rational exploration–exploitation transition. The synergistic effect of these three strategies allows MDOA to achieve a superior dynamic balance between global search and local exploitation.

The main contributions are: (1) MDOA integrates a Beta distribution-based opposition learning strategy, a differential mutation mechanism, and nonlinear parameter control, enhancing DOA’s global search capability, convergence speed, and stability; (2) MDOA is evaluated on CEC2017 and CEC2022 (41 functions) using convergence curves, box plots, Wilcoxon rank-sum tests, and radar charts; (3) MDOA is compared with 11 algorithms (DOA, BKA, BWO, COA, DBO, GoldSA, HHO, PO, PSO, SSA, and TOC); (4) MDOA is applied to five engineering problems: compression spring design, gear reducer weight minimization, rolling bearing optimization, tubular column design, and moisture content prediction of Dendrobium huoshanense using near-infrared spectroscopy with a BP neural network.

The paper is organized as follows. Section 2 describes the original DOA and the proposed MDOA. Section 3 presents the experimental results and analysis. Section 4 evaluates MDOA on engineering problems. Section 5 concludes the paper.

## 2. Proposed Methodology

### 2.1. Original Dhole Optimization Algorithm

DOA is inspired by the tracking, encircling, and cooperative attack behaviors of dhole packs. In the exploration phase, it introduces a prey location integrating global and local optimal solutions, employing two modes: a search phase with random perturbations and an encirclement phase using relative positional differences. In the exploitation phase, it simulates hunting and attacking behaviors, adaptively selects approaches based on prey strength, and uses exponential scaling with sine–cosine perturbations for fine-grained updates. However, DOA suffers from a limited exploration range, insufficient population diversity, and weak later-stage convergence.

### 2.2. Modified Dhole Optimization Algorithm (MDOA)

Based on DOA, MDOA introduces three strategies: BD-OBL enhances diversity and search range; the DE/rand-to-best/1 differential mutation improves approximation and perturbation capability; and the nonlinear parameter *c* smooths the exploration–exploitation transition. Unlike DOA’s single update mode, MDOA adaptively switches update methods, significantly enhancing global search capability and stability.

#### 2.2.1. Algorithm Initialization

In MDOA, the initial population is generated uniformly within the search space. Let the population size be N, the problem dimension be dim, and the search bounds be lb and ub. The position of the *i*-th individual in the *j*-th dimension is defined by Equation (1):

(1) $$X(i,j)=lb_{j}+\mathrm{rand}\times (ub_{j}-lb_{j}),\;j=1,2,\ldots ,\dim$$

This ensures a uniform distribution and provides favorable initial conditions.

#### 2.2.2. Exploration Phase

During the exploration phase, MDOA simulates the hunting behavior of dhole packs. Two key variables are defined: the number of members $N_{hunt}$ (a random integer between 5 and 20, see Equation (2)) and the hunting time $T_{hunt}$ (see Equation (3)), which decreases as the search progresses to simulate accelerated convergence. Here, $\mathrm{EF}\in \left[0,1\right]$ is an environmental factor that affects hunting success, with a larger value indicating more favorable conditions.

(2) $$N_{hunt}=\mathrm{round}(5+\mathrm{rand}\times 15)$$

(3) $$T_{hunt}={\left(\frac{1}{1+e^{\left(-0.5\left(N_{hunt}-25\right)\right)}}\right)}^{2}\times \mathrm{EF}$$

A target position $P_{\mathrm{target}}$ is constructed as the average of the local best $P_{local}$ and the global best $P_{global}$, as shown in Equation (4):

(4) $$p_{\mathrm{target}}=\left(p_{local}+p_{global}\right)/2$$

When a random vocalization value is less than 0.5:

If $N_{hunt}<10$, individuals approach the prey using an adaptive contraction coefficient $C_{2}$, as shown in Equation (5):

(5) $$X_{i,j}^{t+1}=X_{i,j}+C_{2}\times \mathrm{rand}\times \left(p_{\mathrm{targrt},j}-X_{i,j}\right)$$

In the original DOA, $C_{2}$ decreases linearly. To address this, MDOA introduces a nonlinear decay strategy, as shown in Equation (6):

(6) $$C_{2}=1-{\left(\frac{t}{T}\right)}^{2}$$

where t denotes the current iteration and T denotes the maximum number of iterations. In MDOA, $C_{2}$ is embedded into the position update process to regulate the search step and control the transition from exploration to exploitation. At the early stage, $C_{2}$ remains relatively large, allowing individuals to perform broader global exploration and maintain population diversity. As the iteration proceeds, $C_{2}$ decreases nonlinearly and approaches zero, which weakens the disturbance intensity and encourages refined local exploitation around promising regions. Compared with the original linear control strategy, the proposed nonlinear scheme provides a smoother early search and a more concentrated late-stage search, thereby improving convergence efficiency and reducing the risk of premature convergence.

If $N_{hunt}\geq 10$, the dhole pack surrounds the prey, as shown in Equation (7):

(7) $$X_{i,j}^{t+1}=X_{i,j}^{t}-X_{z,j}^{t}+p_{\mathrm{target},j}$$

where z is a randomly selected index, with z≠i.

#### 2.2.3. Attack Phase

After the searching and encirclement phase, when the vocalization value exceeds 0.5, the dhole pack enters the attack phase. The attack strategy depends on the prey strength factor η, defined in Equation (8):

(8) $$\eta =C_{3}\times \mathrm{rand}\times \left(fitness_{i}/fitness_{p}\right).$$

where $fitness_{i}$ is the fitness of the current individual and $fitness_{p}$ is the fitness of the target position.

When η>2 (difficult prey), the prey is large or strong, requiring multiple attacks. A weakened prey vector is first defined using Equation (9):

(9) $$w_{p}=e^{\left(-\frac{1}{\eta }\right)}p_{local}$$

where $P_{local}$ is the current population’s best position. The position is then updated using a sine–cosine hybrid perturbation approach, as shown in Equation (10):

(10) $$X_{i,j}^{t+1}=X_{i,j}^{t}+w_{p}T_{hunt}\times \left(\cos\left(2\pi \times \mathrm{rand}\right)-\sin\left(2\pi \times \mathrm{rand}\right)\times w_{p}\times T_{hunt}\right)$$

When η≤2 (weak prey), the prey is small or already weakened. Individuals adopt a concentrated attack strategy, as shown in Equation (11):

(11) $$X_{i,j}^{t+1}=\left(X_{i,j}^{t}-p_{global,j}\right)\times T_{hunt}+T_{hunt}\times rand(1,\dim)\times X_{i,j}^{t}$$

This accelerates convergence toward the optimal solution. The weak prey strategy further affects the simulation results by regulating the movement amplitude during the exploitation stage. When the prey is regarded as weak, individuals perform a more concentrated search around promising regions, which helps improve local refinement and convergence speed. Meanwhile, this strategy can reduce overly aggressive movement toward the current best solution, thereby decreasing the risk of premature convergence and improving the stability of MDOA on multimodal, hybrid, and composite functions.

#### 2.2.4. DE/Rand-to-Best/1 Differential Mutation Mechanism

When η>2, the attack mechanism alone may cause population diversity loss and increase the risk of local optima. To address this, a differential mutation mechanism is introduced after Equation (10), applying a secondary perturbation as shown in Equation (12):

(12) $$X_{i}^{t+1}=\left\{\begin{array}{ll} X_{i}^{t}+F\times \left(p_{local}-X_{i}^{t}\right)+F\times \left(X_{r1}^{t}-X_{r2}^{t}\right), & \mathrm{rand}>0.5\\ X_{i}^{t}, & \mathrm{otherwise} \end{array}\right.$$

where $X_{r1}^{t}$ and $X_{r2}^{t}$ are two distinct randomly selected individuals and both F and rand are random numbers in $\left[0,1\right]$. This mechanism enhances population diversity while maintaining convergence, reducing the likelihood of local optima and improving overall search performance.

#### 2.2.5. Beta Distribution-Based Opposition Learning Strategy

To enhance population diversity and improve the ability of MDOA to escape local optima, a Beta distribution-based opposition learning (BD-OBL) strategy is introduced after the position update stage. Unlike conventional opposition-based learning, BD-OBL generates adaptive asymmetric opposite candidates according to the current population distribution, thereby improving the flexibility of candidate generation [34].

Suppose that the candidate population generated after the position update stage is denoted as $X^{t,new}$, where $X_{i}^{t,new}$ represents the *i*-th candidate solution. The dynamic boundary vectors $d_{min}$ and $d_{max}$ are constructed according to the minimum and maximum values of the current candidate population in each dimension. To measure the distribution state of the population, the normalized distance between individuals is calculated, as shown in Equation (13).

(13) $$\delta_{i,k}=\frac{1}{\sqrt{D}}{\left\|\left.\frac{X_{i}^{t,new}-X_{k}^{t,new}}{d_{\max}-d_{\min}}\right\|\right.}_{2}$$

where D is the problem dimension and the division operation is performed element-wise. Based on the normalized distance, the crowding distance of each individual and the average population diversity are calculated, as shown in Equation (14).

(14) $$CD_{i}=\underset{k\neq i}{\min}\delta_{i,k},normDiv=\frac{1}{N}\sum_{i=1}^{N}CD_{i}$$

where $CD_{i}$ denotes the minimum normalized distance between the *i*-th individual and the remaining individuals and normDiv represents the average diversity of the current population.

According to the diversity state, concave or convex sampling modes are selected to generate adaptive opposite candidates. The opposite candidate generated by BD-OBL is defined in Equation (15).

(15) $$X_{i}^{opp}=\left(d_{max}-d_{min}\right)\cdot \mathrm{Beta}\left(\alpha_{i},\beta_{i}\right)+d_{min}$$

where $X_{i}^{opp}$ denotes the opposite candidate of the *i-th* individual, $\mathrm{Beta}\left(\alpha_{i},\beta_{i}\right)$ is a Beta-distributed random vector independently sampled in each dimension, and $\alpha_{i}$ and $\beta_{i}$ are determined by the selected sampling mode and the current population diversity.

After $X_{i}^{opp}$ is generated, boundary control is applied to keep it within the feasible search range. Then, a greedy selection mechanism is performed. If the opposite candidate obtains a better fitness value than the original candidate, it replaces the original solution. If it further improves the current global best solution, the global best position is updated. Therefore, BD-OBL improves population diversity without increasing the population size and enhances the ability of MDOA to escape local optima.

**Figure 1:**
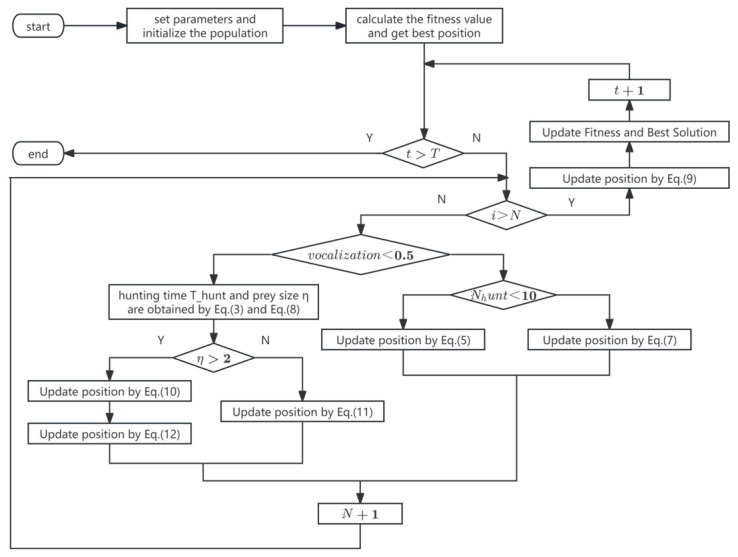
Flowchart of MDOA.

By introducing the BD-OBL strategy, MDOA adaptively adjusts the opposition search range according to the current population distribution. This mechanism enhances local perturbation while maintaining sufficient solution-space coverage, thereby improving the global search performance and convergence stability of MDOA. The pseudocode of the proposed MDOA is presented in Algorithm 1. Figure 1, it presents the flowchart of MDOA.
**Algorithm 1** The algorithm of MDOAStart MDOA1. Input all information of optimization algorithm.2. Set the Dholes (N) and the total iterations (T) numbers.3. Initialize Dholes population.4. Calculate fitness value and get the best solutions.5. t = 16. while t < T 7. Define $N_{\mathrm{hunt}}$ using Equation (2) 8. Define a prey using Equation (4) 9. For i = l:N  10. Vocalization = rand  11. If vocalization < 0.5   12. If $N_{\mathrm{hunt}}$ < 10    13. Compute the contraction coefficient $C_{2}$ using the modified Equation (6).    14. Update position toward the prey using Equation (5)   15. Else    16. Doles perform encircle stage using Equation (7)   17. End If  18. Else   19. hunting time $T_{\mathrm{hunt}}$ and prey size η are obtained by Equations (3) and (8)   20. If η > 2    21. Dholes injure the prey by Equation (9)    22. Dholes kill the prey by Equations (10) and (12)   23. Else    24. Dholes kill the prey by Equation (11)   25. End If  26. End If 27. End for 28. Update population and global best using Beta-distribution opposition-based learning by Equation (13). 29. Update fitness and the best possible solution is available so far. 30. t = t + 131. End whileEnd MDOA

#### 2.2.6. Computational Complexity Analysis

To clarify the computational cost of MDOA, the time complexity of the proposed algorithm is analyzed in this section. Let N, D, T and $C_{f}$ denote the population size, problem dimension, maximum number of iterations, and computational cost of one fitness evaluation, respectively. In the initialization stage, generating the initial population requires $O\left(ND\right)$, and evaluating the initial population requires $O\left(NC_{f}\right)$.

In each iteration, the basic position update, weak prey strategy, DE/rand-to-best/1 mutation mechanism, and BD-OBL strategy mainly involve population-level vector operations, with a computational cost of $O\left(ND\right)$. In addition, the BD-OBL strategy generates opposite candidates and evaluates their fitness values, resulting in an additional cost of $O\left(NC_{f}\right)$. Therefore, the overall time complexity of MDOA can be simplified as $O\left(TN(D+C_{f})\right)$.

Compared with the original DOA, MDOA introduces additional mutation, opposition learning, and greedy selection operations. These operations increase the computational cost by a constant factor but do not change the dominant complexity order of the algorithm. Compared with other population-based metaheuristic algorithms used in this study, MDOA maintains the same dominant complexity order because these algorithms generally update and evaluate N candidate solutions over T iterations. Therefore, MDOA improves search capability while maintaining acceptable computational complexity.

## 3. Experimental Results and Analysis

### 3.1. Experimental Environment

The simulations were conducted on Windows 11 with a 13th-generation Intel Core i7 processor at 2.40 GHz and 32 GB of memory. All algorithms were implemented in MATLAB R2023a. For the CEC2017 and CEC2022 benchmark experiments, all algorithms used the same population size of 30, a maximum number of iterations of 1000, and 30 independent runs. The termination criterion was reaching the maximum number of iterations, and no additional early-stopping condition was used. Therefore, the convergence behavior of all algorithms was compared under the same iteration budget. The reported statistical results were calculated based on the 30 independent runs.

### 3.2. CEC2017 Test Functions

Section 3.2 evaluates the performance of MDOA and 11 other algorithms (DOA, TOC, BKA, COA, SSA, PO, PSO, BWO, GoldSA, DBO, and HHO) using the CEC2017 test functions.

The CEC2017 benchmark suite comprises 29 functions (F1 and F3–F30), including unimodal (F1 and F3), multimodal (F4–F10), hybrid (F12–F20), and composite functions (F21–F30). With rotation, shifting, and variable coupling, it systematically evaluates an algorithm’s global search, local exploitation, and robustness [35].

#### 3.2.1. Analysis of Key Indicators for CEC2017

Table 1 presents the average fitness results for F1–F10. Based on the ranking rule (mean first, then standard deviation), the performance of MDOA is quantitatively analyzed across different test functions.

As shown in Table 1, MDOA achieves the lowest average fitness on F4, F5, F6, and F8, ranking first on these multimodal functions (F4 and F5 have numerous local extrema), demonstrating a strong global search capability and an ability to escape local optima.

On the remaining functions, MDOA maintains a top overall ranking. It ranks second on F1, F3, and F7, second only to algorithms with stronger exploitation; fourth on F9, close to top-ranked methods; and sixth on F10, still stable compared to most competitors.

Overall, MDOA exhibits a pattern of “leading on multiple functions and consistently top-ranked on others” across F1–F10. Its rankings are concentrated in the top positions, reflecting good robustness and comprehensive optimization capability. The proposed improvement strategy effectively enhances search performance across most test scenarios while maintaining balanced performance.

Table 2 presents the average fitness results for hybrid functions F11–F20. Based on the same ranking rule, the performance of MDOA is further analyzed.

As shown in Table 2, MDOA achieves the lowest average fitness on nine hybrid functions (F11–F17, F19, and F20), ranking first overall. Hybrid functions combine characteristics of multiple basic functions across different dimensions, creating complex search spaces that demand strong global search and local exploitation. MDOA consistently obtains superior solutions, demonstrating strong adaptability to problems with coupled variables and uneven search characteristics.

On F18, MDOA ranks second, slightly behind DOA but close to the optimal result, and significantly outperforms other comparative algorithms, showing strong competitiveness and stability.

Overall, MDOA exhibits the pattern of “optimal on most hybrid functions and close to optimal on the remaining few,” fully demonstrating its robustness and comprehensive optimization capability. This further validates the effectiveness of the proposed improvement strategy in addressing high-dimensional, strongly coupled hybrid optimization problems.

As shown in Table 3, MDOA achieves the lowest average fitness on composite functions F21, F23, F24, F25, F27, and F28, ranking first overall. Composite functions weight and superimpose multiple basic functions with rotation, translation, and variable coupling, creating highly complex search spaces. Achieving optimal results for such functions demonstrates MDOA’s strong global search and stable local exploitation capabilities. On F26, F29, and F30, MDOA consistently ranks second, behind DOA on F26 and F29, and closely following the optimal algorithm SSA on F30. Although not the best, the gap is small, and its overall performance remains high, showing good stability and competitiveness. On F22, MDOA does not gain an advantage, indicating that other algorithms have greater advantages on this specific combinatorial search structure.

Overall, MDOA exhibits the pattern of “optimal on most composite functions, close to optimal on some, and not dominant on a few,” fully demonstrating the effectiveness and robustness of the proposed improvement strategy in addressing high-complexity, multimodal, and strongly coupled search problems.

#### 3.2.2. Convergence Analysis of CEC2017 Test Functions

**Figure 2:**
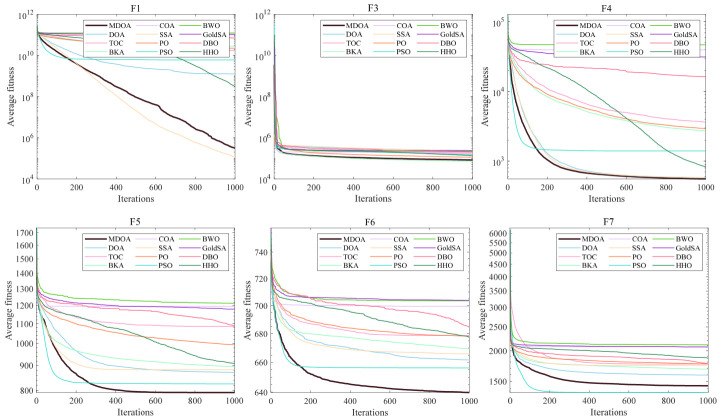

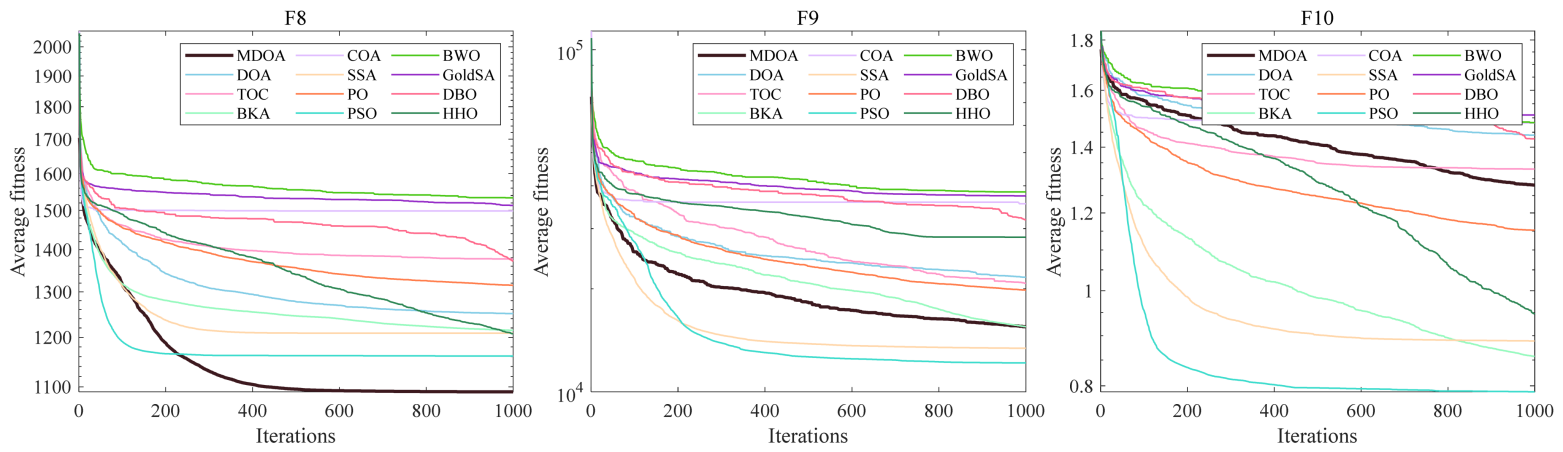
Average convergence curves of F1–F10 for the CEC2017 test functions.

Figure 2 shows the average convergence curves for F1–F10. MDOA exhibits fast initial convergence and smooth optimization on most functions. On F4, F5, F6, and F8, MDOA declines fastest and converges to the lowest level, demonstrating advantages in avoiding premature convergence and enhancing local exploitation. On F1 and F3, MDOA remains at a lower level, though slightly inferior to SSA on F1 and close to optimal on F3. On F7, F9, and F10, MDOA shows smooth convergence but does not reach the optimum; it remains top-tier on F7 and F9, while other algorithms perform better on F10. Overall, MDOA achieves rapid convergence and stable optimization on most F1–F10 functions, validating the effectiveness of the proposed improvement strategy.

**Figure 3:**
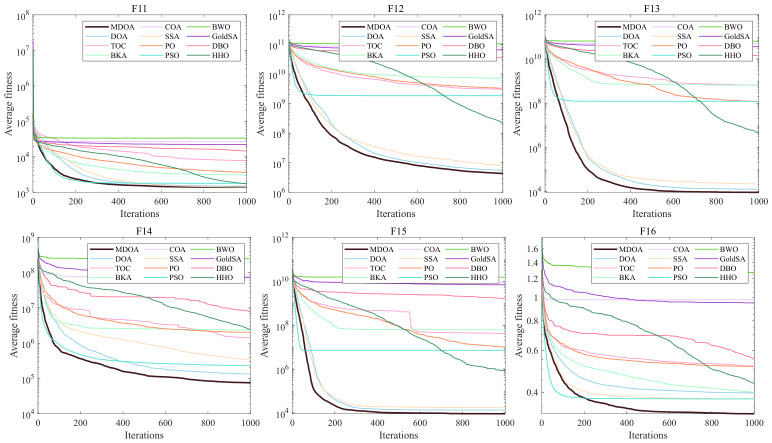

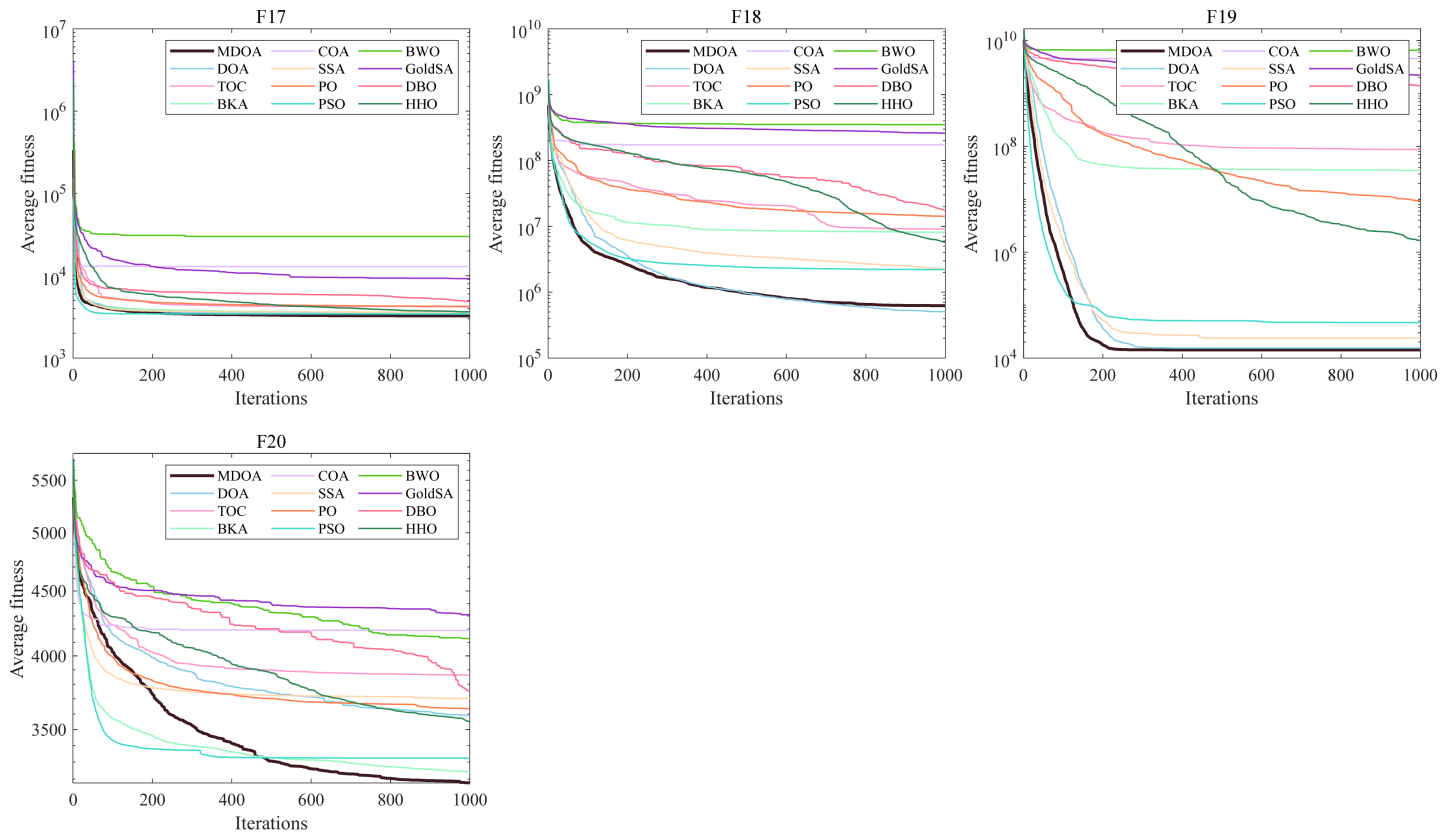
Average convergence curves of test functions F11–F20 for CEC2017.

Figure 3 presents the average convergence curves of the CEC2017 hybrid test functions F11–F20. It can be observed that, for this category of functions, MDOA demonstrates a relatively fast initial convergence and a smoother convergence process on most test functions, reflecting its strong global search capability and search stability.

MDOA demonstrates fast initial convergence and a smooth process on most functions. On F11–F17 and F20, MDOA drops rapidly in early iterations and continuously improves, ultimately converging to the lowest level. Notably, on F13 and F15, MDOA shows significant advantages in both descent speed and final accuracy. On F18, MDOA and DOA show similar mid–late convergence, both outperforming other algorithms, though DOA achieves a slightly lower level. MDOA remains smooth and in the leading group, demonstrating good stability. On F19, MDOA’s descent is not the fastest initially, but it is the first to reach the lowest convergence plateau and maintains the lowest level with minimal fluctuations, showcasing “stable late-stage dominance.” Overall, MDOA exhibits two complementary patterns on hybrid functions: a rapid initial decline followed by sustained optimization on most functions, and stable late-stage dominance on F19. This suggests that the multi-strategy fusion mechanism adaptively adjusts search behavior across different landscapes, maintaining stable and efficient optimization.

**Figure 4:**
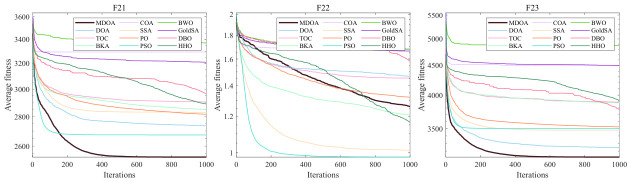

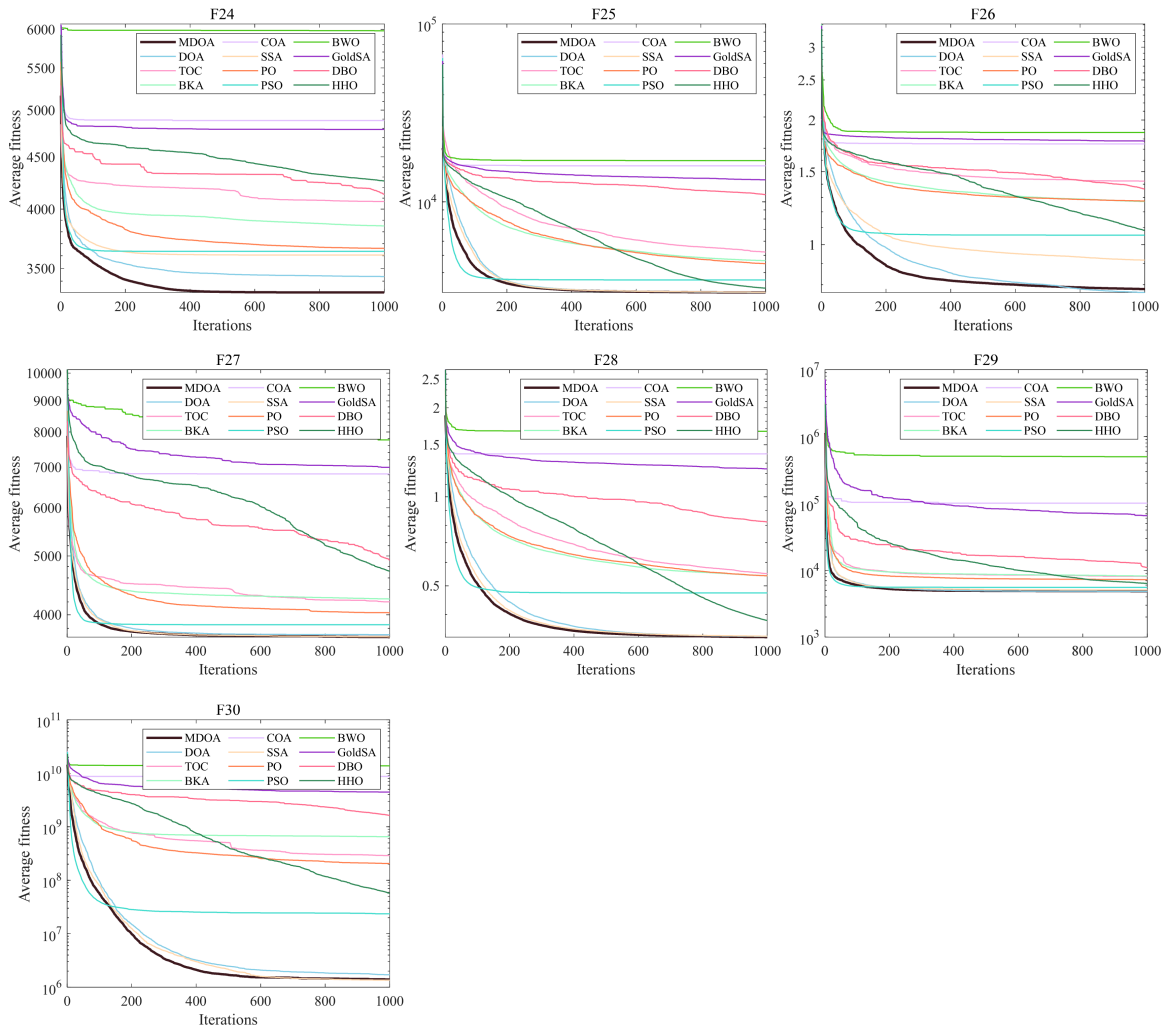
Average convergence curves for test functions F21–F30.

Figure 4 presents the average convergence curves of the CEC2017 composite test functions F21–F30, illustrating the convergence behavior characteristics of various comparative algorithms in highly complex search spaces. It can be observed that, within this category of composite functions, there are significant differences in the convergence trends among different algorithms. MDOA demonstrates a relatively smooth and continuous declining process on most test functions, reflecting its good search stability.

On F21, F23, and F24, MDOA drops rapidly in early iterations and remains at a low level, demonstrating strong global search capability. On F25, F27, and F28, MDOA maintains a continuous optimization trend in mid–late stages, showing good local exploitation and stability, while other algorithms flatten out earlier. On F22, MDOA declines steadily but is inferior to PSO, SSA, and BKA in convergence speed and final level. On F26, MDOA’s convergence is smooth but slightly less accurate than DOA. On F29 and F30, MDOA ranks second, showing strong stability and competitiveness.

Overall, MDOA maintains a stable and effective optimization process on most composite functions, achieving superior convergence on the majority. Although not advantageous on a few functions, its overall convergence remains steady and reliable, validating its adaptability to highly complex combinatorial optimization problems.

The fast initial convergence of MDOA is mainly attributed to nonlinear parameter control and the DE/rand-to-best/1 mutation mechanism, which enable individuals to rapidly approach promising regions while maintaining population diversity. In the later stage, the gradual reduction of the search step and the adaptive opposite candidates generated by the BD-OBL strategy reduce unnecessary oscillations and improve local refinement. Therefore, MDOA shows fast initial convergence and a relatively smooth optimization process on most CEC2017 functions.

#### 3.2.3. Stability Analysis of CEC2017

In addition to average performance, the consistency of results across multiple independent runs is crucial for evaluating algorithm reliability. This paper employs box plots to visualize result distributions. The box height represents the interquartile range (a narrower box indicates higher stability), the whiskers reflect the overall fluctuation range, and outliers indicate extreme results. Fewer outliers suggest greater robustness. Thus, box plots enable a thorough evaluation of algorithm stability.

**Figure 5:**
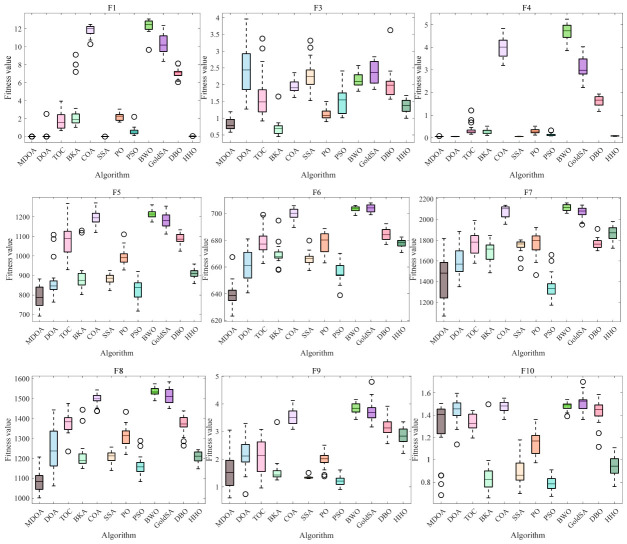
Box plots of functions F1–F10 for CEC2017.

Figure 5 shows box plots for F1–F10. On unimodal functions (F1, F3, and F4), MDOA exhibits the most compact boxes, medians close to optima, and few outliers, indicating strong convergence capability and stability. On multimodal functions (F5–F8), box heights increase for all algorithms, reflecting greater search difficulty, but MDOA maintains a small fluctuation range and superior median distribution, demonstrating good robustness. On complex functions (F9 and F10), MDOA shows concentrated distributions with small fluctuations, effectively balancing global exploration and local exploitation. In summary, MDOA demonstrates superior stability and comprehensive performance on most test functions.

**Figure 6:**
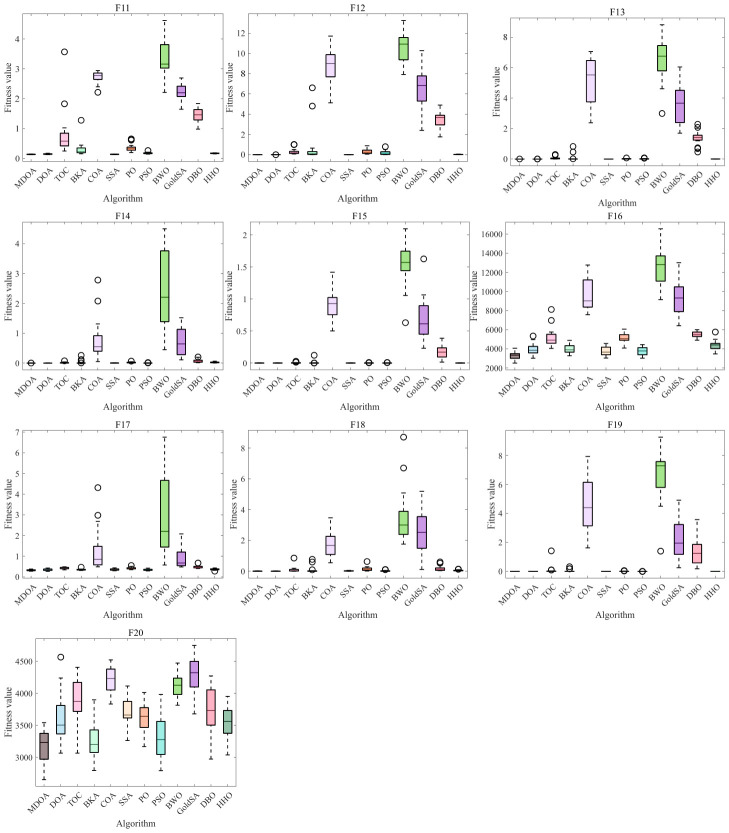
Box plots of functions F11–F20 for CEC2017.

Figure 6 shows box plots for hybrid functions F11–F20. On F11–F15 and F17–F19, MDOA’s box height is nearly zero, indicating highly concentrated results and strong stability. The narrow box width and few outliers suggest small convergence fluctuations. On F16 and F20, result differences among algorithms are more pronounced, but MDOA still maintains a relatively small box width and more stable fitness distribution, demonstrating good robustness and convergence reliability.

In summary, MDOA exhibits stable optimization performance on F11–F20, maintaining consistent convergence behavior across multiple runs.

**Figure 7:**
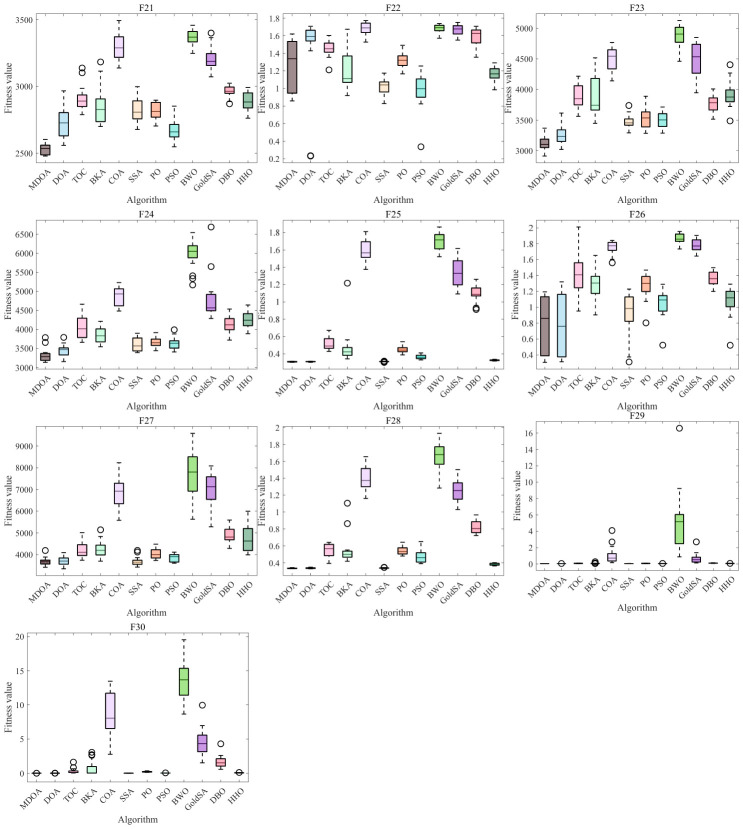
Box plots of functions F21–F30 for CEC2017.

Figure 7 shows box plots for composite functions F21–F30. On most F21–F28 tests, MDOA exhibits low median values and narrow box widths, indicating concentrated results and small convergence fluctuations. On F22 and F26, MDOA’s median is higher than some algorithms, showing no clear advantage, but its distribution remains stable. In contrast, BWO shows significant volatility with larger box heights and longer whiskers. On F29 and F30, most algorithms (excluding BWO) achieve results close to the optimum, and MDOA maintains relatively narrow box widths and stable distribution characteristics.

In summary, MDOA demonstrates overall stable optimization performance on composite functions F21–F30 and exhibits good robustness in complex search spaces.

Overall, MDOA exhibits concentrated distributions with narrow box widths and few outliers across most F1–F30 test functions, indicating good stability and consistency across multiple independent runs, as well as robustness with few significant abnormal fluctuations. Moreover, MDOA maintains stable convergence performance on unimodal, multimodal, hybrid, and composite functions, reflecting its adaptability across diverse optimization scenarios. In summary, MDOA demonstrates reliable optimization performance and good stability on all 29 CEC2017 test functions.

#### 3.2.4. Statistical Analysis of CEC2017

Table 4 presents the Wilcoxon rank-sum test results. At the significance level *α* = 0.05, MDOA shows significant differences from most comparative algorithms (*p* < 0.05) on the vast majority of test functions, demonstrating its strong competitive advantages. As MDOA is an improvement of DOA, their differences are smaller on some functions, but MDOA still achieves better overall performance. In summary, MDOA significantly outperforms most algorithms and surpasses DOA, confirming the effectiveness of the proposed improvement strategy.

**Figure 8:**
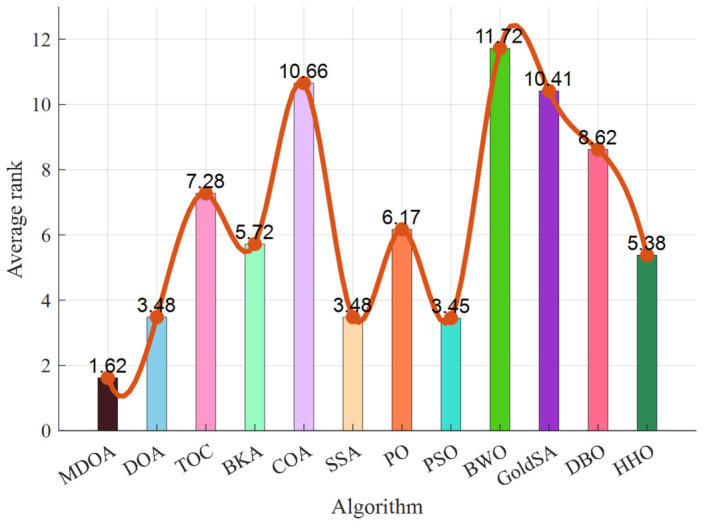
Average ranking chart for CEC2017.

Figure 8 presents the average ranking of each algorithm on CEC2017. MDOA achieves the best average ranking of 1.62, significantly outperforming the original DOA (3.48) and all other compared algorithms, confirming the effectiveness of the proposed improvement strategy.

In summary, MDOA achieves the lowest average ranking, demonstrating the best overall optimization performance and validating the effectiveness of the proposed improvement strategies.

#### 3.2.5. Analysis of the Radar Chart for CEC2017

**Figure 9:**
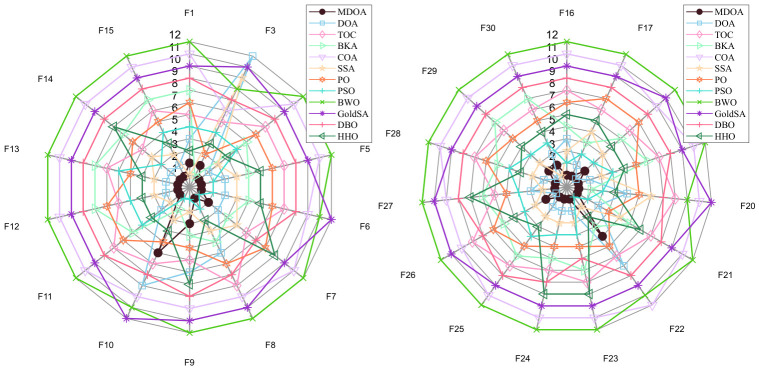
Radar chart for CEC2017 test functions.

Figure 9 presents radar charts of ranking distributions for F1–F15 and F16–F30. MDOA (in black) is distributed closer to the center, indicating higher rankings on most functions and superior overall performance. In contrast, BWO, GoldSA, and DBO are closer to the outer circle, showing lower rankings.

On the more complex F16–F30, MDOA’s distribution remains concentrated with minimal volatility, demonstrating stable performance across different function types. Compared to the original DOA, MDOA ranks higher on most functions, confirming that the proposed improvement strategy effectively enhances search capability and optimization performance.

### 3.3. CEC2022 Test Functions

The CEC2022 benchmark comprises 12 single-objective functions, categorized into unimodal (F1–F4), multimodal (F5–F10), and composite functions (F11–F12) [36]. Unimodal functions test convergence speed and local exploitation; multimodal functions assess global exploration capability; composite functions create complex problem structures for comprehensive evaluation. Experiments are conducted in 20 dimensions.

#### 3.3.1. Analysis of Key Indicators for CEC2022

Table 5 presents the statistical results for CEC2022. Overall, MDOA ranks first on 7 out of 12 functions and remains within the top three on the remaining ones, demonstrating outstanding overall performance. On unimodal functions (F1–F4), MDOA achieves the lowest average fitness on F1–F3 and ties with the optimal PSO on F4 (ranking second due to a slightly larger standard deviation), confirming its strong local exploitation capability. On multimodal functions (F5–F10), MDOA achieves the best average fitness on F5, F7, and F8 and ranks within the top three across all multimodal functions, showcasing effective global exploration. On composite functions (F11–F12), MDOA ranks first on F12 and second on F11, reflecting strong adaptability and robustness. Additionally, MDOA achieves relatively low standard deviations on most functions, indicating good stability across multiple independent runs, with notable advantages on both unimodal and typical multimodal problems.

#### 3.3.2. Convergence Analysis of CEC2022 Test Functions

**Figure 10:**
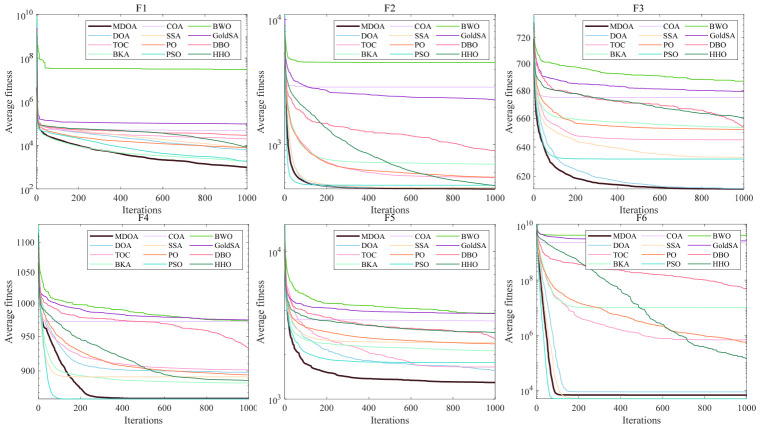

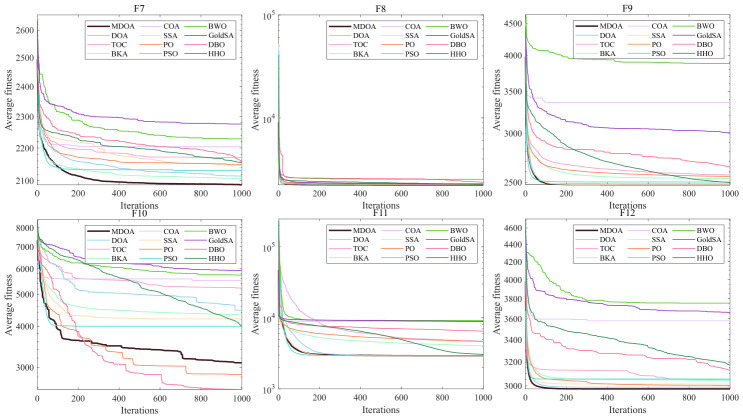
Average convergence curves of F1–F12 for the CEC2022 test functions.

To further analyze the search behavior and convergence characteristics of various algorithms, Figure 10 presents the average convergence curves for CEC2022.

On unimodal functions (F1–F3), MDOA rapidly reduces fitness in early iterations and maintains a stable decline, achieving superior results, while competitors stagnate in mid–late stages. On F4, MDOA shows stable convergence and favorable results. On multimodal functions (F5, F7, and F8), MDOA maintains low fitness throughout and achieves superior results. On F6 and F9, the results are close, but MDOA shows more stable convergence. On F10, MDOA converges fast initially, though DBO ultimately achieves better results. On composite functions (F11–F12), MDOA rapidly reduces fitness and maintains stable optimization, with consistently low fitness on F12.

Overall, MDOA demonstrates fast convergence and stable search on most CEC2022 functions, reflecting a good balance between exploration and exploitation.

The convergence behavior of MDOA on CEC2022 functions further demonstrates the effectiveness of the proposed multi-strategy framework. Nonlinear parameter control and DE/rand-to-best/1 mutation improve early-stage search efficiency, while the reduced search step and BD-OBL-based adaptive candidate generation enhance later-stage stability. This explains why MDOA maintains stable convergence on different types of CEC2022 functions.

#### 3.3.3. Stability Analysis of CEC2022

**Figure 11:**
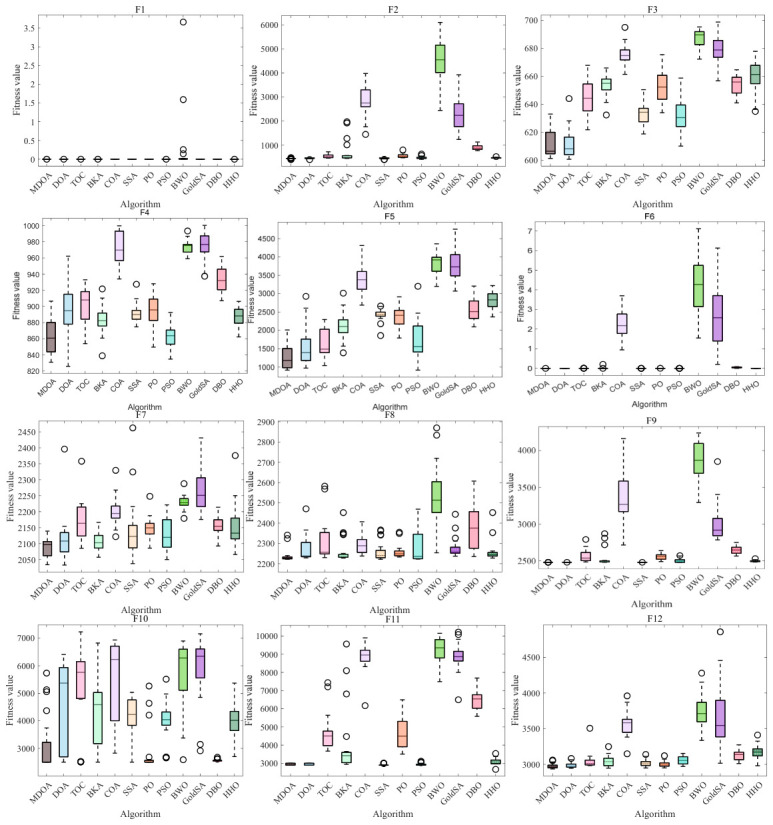
Box plots of functions F1–F12 for CEC2022.

To further evaluate stability under multiple independent runs, Figure 11 presents box plots for CEC2022.

On unimodal functions (F1–F4), MDOA exhibits narrow boxes and low medians, indicating stable superior results across runs. Particularly on F1 and F2, MDOA shows more concentrated distributions, demonstrating good stability and consistency. In contrast, some competing algorithms show wider box spans and outliers, indicating weaker stability. On multimodal functions (F5–F10), MDOA shows narrow boxes and low medians on F5, F7, and F8, maintaining stable performance in complex search spaces. Some competitors show dispersed distributions and multiple outliers, being susceptible to local optima. On F6 and F9, despite close result distributions, MDOA still shows more concentrated solutions, reflecting superior stability. On composite functions (F11–F12), MDOA’s result distributions remain concentrated with small box heights, demonstrating good stability and robustness.

Overall, MDOA exhibits low result dispersion and few outliers on most CEC2022 functions, demonstrating good stability and reliability. Compared to competitors, MDOA not only achieves superior optimization results but also shows a significant advantage in result consistency.

#### 3.3.4. Statistical Analysis of CEC2022

Table 6 presents the Wilcoxon rank-sum test results for CEC2022 (*α* = 0.05). MDOA exhibits significant differences (*p* < 0.05) from TOC, COA, PO, BWO, GoldSA, DBO, and HHO on all test functions and from BKA, PSO, and SSA on most functions, demonstrating its strong statistical superiority. As MDOA improves upon DOA, their differences are smaller on some functions, but MDOA still achieves better overall performance on most functions. In summary, MDOA significantly outperforms most comparative algorithms on CEC2022, further validating the effectiveness and superiority of the proposed algorithm from a statistical perspective.

**Figure 12:**
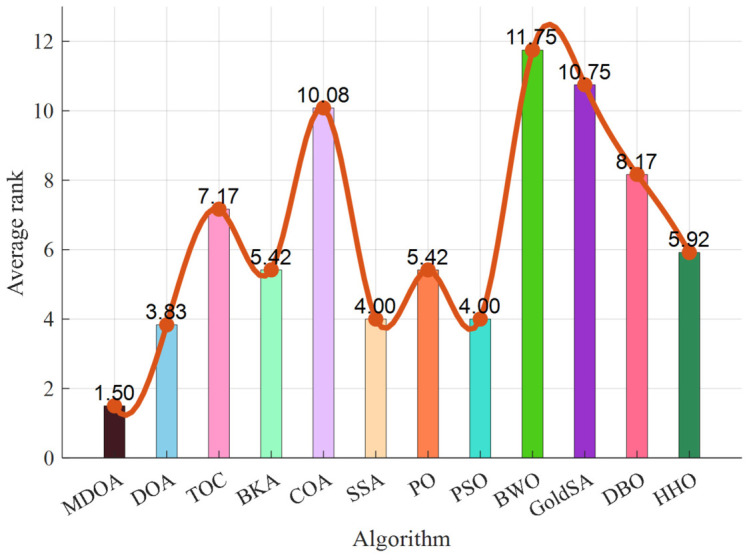
Average ranking chart for CEC2022.

Figure 12 presents the average ranking of each algorithm on CEC2022. MDOA achieves the lowest average ranking of 1.50, significantly outperforming the original DOA (3.83) and all other compared algorithms, confirming the effectiveness of the proposed improvement strategy.

#### 3.3.5. Analysis of the Radar Chart for CEC2022

**Figure 13:**
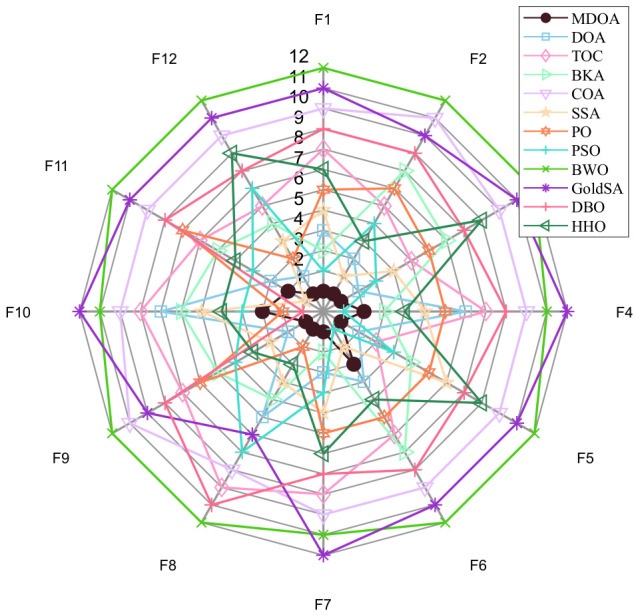
Radar chart for CEC2022 test functions.

To more intuitively display the performance distribution of various algorithms on different test functions, Figure 13 presents radar charts of each algorithm on the CEC2022 test functions. The radar charts can reflect the ranking changes of different algorithms across various test functions, where a closer distance to the center indicates better algorithm performance.

Figure 13 presents radar charts on CEC2022. MDOA is positioned closest to the center, indicating the best and most stable performance across test functions. In contrast, DOA, SSA, and PSO show larger distributions, indicating inferior stability. The radar chart validates that MDOA achieves both good overall performance and favorable stability across different function types.

## 4. Engineering Optimal Design

This section evaluates MDOA on five engineering application problems, including four classical engineering design problems and one moisture content prediction task. These problems feature complex constraints, nonlinearity, and multivariable coupling. Comparative experiments assess the algorithm’s performance in terms of optimization accuracy, stability, and statistical significance. For the four classical engineering design problems in Section 4.1, Section 4.2, Section 4.3 and Section 4.4, all algorithms were evaluated using a population size of 30, a maximum of 1000 iterations, and 20 independent runs.

### 4.1. Design Problem of Compression Springs

**Figure 14:**
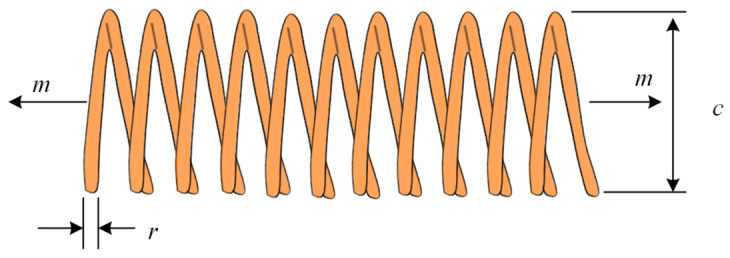
Schematic diagram of spring structure.

The compression spring design problem is a typical constrained engineering optimization problem. Its objective is to minimize the spring weight by optimizing structural parameters while satisfying multiple constraints. A schematic diagram is shown in Figure 14.

This problem involves three decision variables: spring wire diameter $z_{1}$, spring mean diameter $z_{2}$, and number of active coils $z_{3}$. Let $z=\left[z_{1},\;z_{2},\;z_{3}\right]=\left[r,\;c,\;m\right]$. The mathematical model is expressed in Equation (16).

(16) $$\min f(z)=(2+z_{3})z_{2}{z_{1}}^{2}$$

Meanwhile, this design problem needs to satisfy the inequality constraints given by Equation (17):

(17) $$\begin{array}{l} c_{1}(z)=1-\frac{z_{2}^{2}z_{3}}{71785z_{1}^{4}}\leq 0,c_{2}(z)=\frac{4z_{2}^{2}-z_{1}z_{2}}{12566(z_{2}z_{1}^{3}-z_{1}^{4})}+\frac{1}{5108z_{1}^{2}}-1\leq 0\\ c_{3}(z)=1-\frac{140.45z_{1}}{z_{2}^{2}z_{3}}\leq 0,c_{4}(z)=\frac{z_{1}+z_{2}}{1.5}-1\leq 0 \end{array}$$

The range of values for the design variables is shown in Equation (18):

(18) $$0.05\leq z_{1}\leq 2.00,0.23\leq z_{2}\leq 1.30,2.00\leq z_{3}\leq 15.00$$

**Figure 15:**
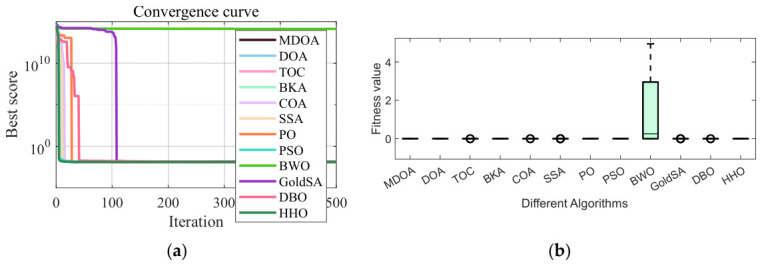
(**a**) Average convergence curve for the compression spring problem. (**b**) Box plot for the compression spring problem.

Comparative experiments were conducted with a population size of 30, a maximum of 1000 iterations, and 20 independent runs. As shown in Table 7, MDOA achieves the optimal value of 0.012665 with design variables $z=\left(0.051605,0.354699,\;11.4083\right)$, outperforming all compared algorithms. The convergence curve in Figure 15a shows that MDOA rapidly reduces the objective function in early iterations and stabilizes quickly, demonstrating fast convergence and good search ability.

MDOA achieves an average value of 0.01283 and a standard deviation of 0.000195, significantly smaller than most comparative algorithms, demonstrating good stability. The box plot in Figure 15b shows that MDOA’s results are concentrated with almost no outliers, while some algorithms exhibit significant fluctuations. The Wilcoxon rank-sum test results in Table 8 show significant differences (*p* < 0.05) between MDOA and all compared algorithms, confirming its statistical superiority. These results further validate the effectiveness of MDOA in the compression spring design problem.

### 4.2. Minimization Problem of Reducer Weight

**Figure 16:**
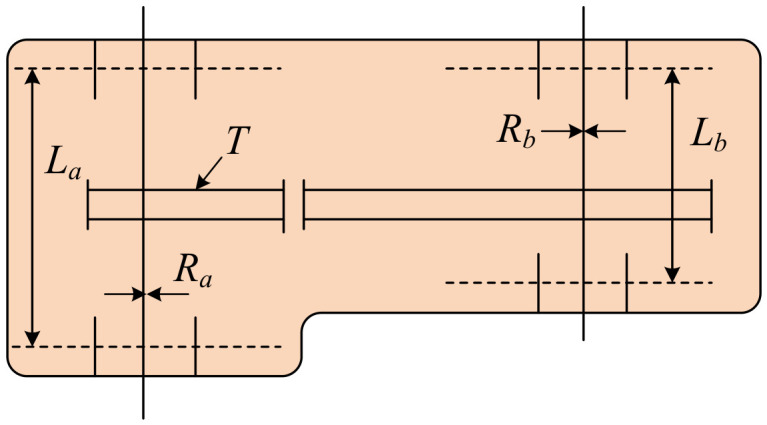
Schematic diagram of reducer problem.

The speed reducer weight minimization problem is a classical constrained engineering optimization problem. A schematic diagram is shown in Figure 16.

The objective is to minimize the weight by optimizing structural parameters while satisfying strength and dimensional constraints. Seven design variables are denoted as $z=\left[z_{1},z_{2},z_{3},z_{4},z_{5},z_{6},z_{7}\right]=[W,P,T,L_{a},L_{b},R_{a},R_{b}]$. Here, W represents the gear face width, P represents the modulus, T represents the number of teeth, $L_{a}$ and $L_{b}$ represent the bearing spacings, and $R_{a}$ and $R_{b}$ represent the shaft diameters. The objective function of this problem is shown in Equation (19):

(19) $$\begin{array}{l} f(z)=0.7854z_{1}{z_{2}}^{2}(3.3333{z_{3}}^{2}+14.9334z_{3}-43.0934)-1.508z_{1}({z_{6}}^{2}+{z_{7}}^{2})+\\ 7.4777({z_{6}}^{3}+{z_{7}}^{3})+0.7854(z_{4}{z_{6}}^{2}+z_{5}{z_{7}}^{2}) \end{array}$$

This optimization problem needs to satisfy the constraint condition in Equation (20):

(20) $$\begin{array}{l} c_{1}(z)=-z_{1}{z_{2}}^{2}z_{3}+27\leq 0,c_{2}(z)=-z_{1}{z_{2}}^{2}{z_{3}}^{2}+397.5\leq 0,c_{3}(z)=-z_{2}{z_{6}}^{4}z_{3}+1.93{z_{4}}^{3}\leq 0\\ c_{4}(z)=-z_{2}{z_{6}}^{4}z_{3}+1.93{z_{5}}^{3}\leq 0,c_{5}(z)=10{z_{6}}^{-3}\sqrt{16.91\times 10^{6}+{\left(745z_{4}{z_{2}}^{-1}{z_{3}}^{-1}\right)}^{2}}-1100\leq 0\\ c_{6}(z)=10{z_{7}}^{-3}\sqrt{157.5\times 10^{6}+{\left(745z_{5}{z_{2}}^{-1}{z_{3}}^{-1}\right)}^{2}}-850\leq 0,c_{7}(z)=z_{2}z_{3}-40\leq 0\\ c_{8}(z)=5z_{2}-z_{1}\leq 0,c_{9}(z)=z_{1}-12z_{2}\leq 0,c_{10}(z)=1.5z_{6}-z_{4}+1.9\leq 0,c_{11}(z)=1.1z_{7}-z_{5}+1.9\leq 0 \end{array}$$

The range of values for the design variables is shown in Equation (21):

(21) $$2.6\leq z_{1}\leq 3.6,0.7\leq z_{2}\leq 0.8,17\leq z_{3}\leq 18,7.3\leq z_{4},z_{5}\leq 8.3,2.9\leq z_{6}\leq 3.9,5.0\leq z_{7}\leq 5.5$$

Table 9 shows the experimental results. MDOA achieves the optimal value of 2.99 × 10^3^, which is comparable to some algorithms but superior to most. Its small average and standard deviation indicate good robustness across multiple runs.

**Figure 17:**
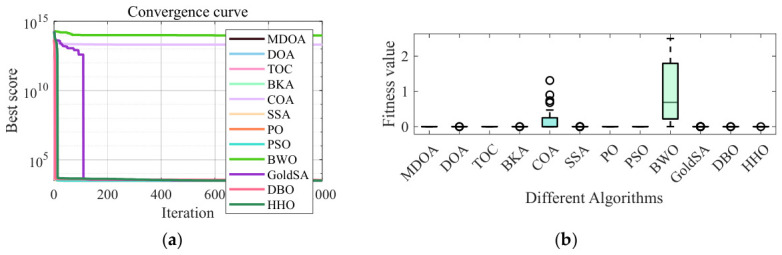
(**a**) Average convergence curve for the reducer weight minimization problem. (**b**) Box plot for the reducer weight minimization problem.

The convergence curve in Figure 17a shows that MDOA rapidly reduces the objective function in early iterations and stabilizes quickly. The box plot in Figure 17b shows that MDOA’s solutions are concentrated with small fluctuations, while some comparative algorithms exhibit greater dispersion.

Table 10 presents the Wilcoxon rank-sum test results, showing significant differences between MDOA and most comparative algorithms, further validating its statistical superiority for this engineering problem.

### 4.3. Optimization Design Problem of Rolling Bearings

**Figure 18:**
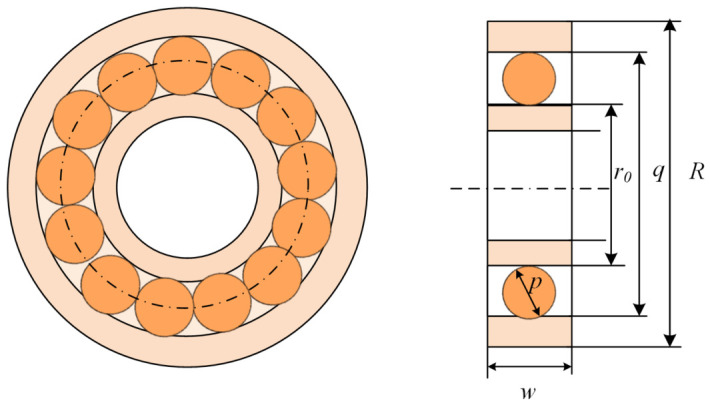
Schematic diagram of rolling bearing problem.

The rolling bearing optimization design is a typical multivariable, strongly constrained engineering problem. Its objective is to maximize the dynamic load-carrying capacity under structural and geometric constraints. Key factors include rolling element dimensions, raceway parameters, and contact characteristics, exhibiting significant nonlinearity and coupling. A schematic diagram is shown in Figure 18. Let the design variable vector be denoted as $x=[z_{1},z_{2},z_{3},z_{4},z_{5},z_{6},z_{7},z_{8},z_{9},z_{10}]=[p,q,m,u,v,w,r,s,t,h]$, where p is the rolling element diameter, q is the pitch diameter, m is the number of rolling elements, and u,v,w,r,s,t,h are structural and contact-related coefficients. The mathematical model of the problem is formulated as Equation (22).

Table 11 shows that MDOA achieves the optimal value of 1.70 × 10^4^, matching DOA, BKA, SSA, and PSO, and outperforming most others. COA and BWO perform significantly worse, indicating they are prone to local optima. MDOA’s mean equals its optimal value, with a standard deviation of only 1.67 × 10^−12^, demonstrating extremely high stability.

(22) $$\begin{array}{l} minimize:\;f(x)=\left\{\begin{array}{ll} \eta m^{2/3}p^{1.8}, & p\leq 25.4\\ 3.647\eta m^{2/3}p^{1.4}, & p>25.4 \end{array}\right.\\ subject\;to:\\ g_{1}(x)=m-\frac{\psi }{2\sin^{-1}(p/q)}-1\leq 0,g_{2}(x)=u(R-r_{0})-2p\leq 0,g_{3}(x)=2p-v(R-r_{0})\leq 0\\ g_{4}(x)=p-sW\leq 0,g_{5}(x)=0.5(R+r_{0})-q\leq 0,g_{6}(x)=q-(0.5+r)(R+r_{0})\leq 0\\ g_{7}(x)=wp-0.5(R-q-p)\leq 0,g_{8}(x)=0.515-t\leq 0,g_{9}(x)=0.515-h\leq 0\\ where:\;\eta =37.91{\left\{1+{\left[1.04{\left(\frac{1-\lambda }{1+\lambda }\right)}^{1.72}{\left(\frac{t(2h-1)}{h(2t-1)}\right)}^{0.41}\right]}^{10/3}\right\}}^{-0.3},\lambda =\frac{p\cos\beta }{q}\\ \psi =2\pi -2\cos^{-1}\left(\frac{{\left(\frac{R-r_{0}}{2}-\frac{3U}{4}\right)}^{2}+{\left(\frac{R}{2}-\frac{U}{4}\right)}^{2}-{\left(\frac{r_{0}}{2}+\frac{U}{4}\right)}^{2}}{2\left(\frac{R-r_{0}}{2}-\frac{3U}{4}\right)\left(\frac{R}{2}-\frac{U}{4}-p\right)}\right)\\ U=R-r_{0}-2p,R=160,\;r_{0}=90,\;W=30,\;W_{i}=R_{i}/p,\;W_{0}=R_{0}/p\\ with\;variable\;bounds:\\ 0.5(R+r_{0})\leq q\leq 0.6(R+r_{0}),0.15(R-r_{0})\leq p\leq 0.45(R-r_{0}),4\leq m\leq 50,0.515\leq W_{i}\leq 0.6\\ 0.515\leq W_{0}\leq 0.6,0.4\leq u\leq 0.5,0.6\leq v\leq 0.7,0.3\leq w\leq 0.4,0.02\leq r\leq 0.4,0.6\leq s\leq 0.85 \end{array}$$

**Figure 19:**
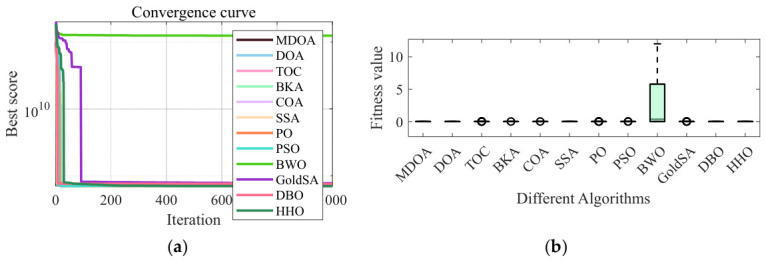
(**a**) Average convergence curve for the rolling bearing optimization problem. (**b**) Box plot for the rolling bearing optimization problem.

The convergence curve in Figure 19a shows rapid reduction and quick stabilization. The box plot in Figure 19b shows that MDOA’s solutions are highly concentrated with almost no outliers, while some competitors exhibit significant dispersion.

Table 12 presents the Wilcoxon rank-sum test results, showing *p* < 0.05 between MDOA and all compared algorithms, further validating its statistical superiority.

### 4.4. Tubular Column Design Problem

The tubular column design problem is a typical structural optimization problem. Its objective is to minimize cost while satisfying strength and stability constraints. It involves only two design variables, average diameter $z_{1}$ and thickness $z_{2}$, characterized by low dimensionality but strong constraint coupling. Let $z=\left[z_{1},z_{2}\right]=\left[d,h_{\omega }\right]$, where d represents the average diameter of the tubular column and $h_{\omega }$ represents the column wall thickness. The optimization model can be expressed as Equation (23):

(23) $$\begin{array}{l} \min f(z)=9.8Dh_{\omega }+2D,c_{1}(z)=\frac{Q}{\pi Dh_{\omega }f_{s}}-1\leq 0,\\ c_{2}(z)=\frac{8QH^{2}}{\pi^{3}YDh_{\omega }(D^{2}+h_{\omega }^{2})}-1\leq 0,c_{3}(z)=\frac{2.0}{D}-1\leq 0,\\ c_{4}(z)=\frac{D}{14}-1\leq 0,c_{5}(z)=\frac{0.2}{h_{\omega }}-1\leq 0,c_{6}(z)=\frac{h_{\omega }}{8}-1\leq 0\\ Subject\;to:2\leq D\leq 14,\;0.2\leq h_{\omega }\leq 0.8 \end{array}.$$

Among them, the material parameters, namely, the yield strength $f_{s}=500\;kgf/cm^{2}$, elastic modulus $Y=0.85\times 10^{6}\;kgf/cm^{2}$, external load Q, and length L, are known constants.

**Figure 20:**
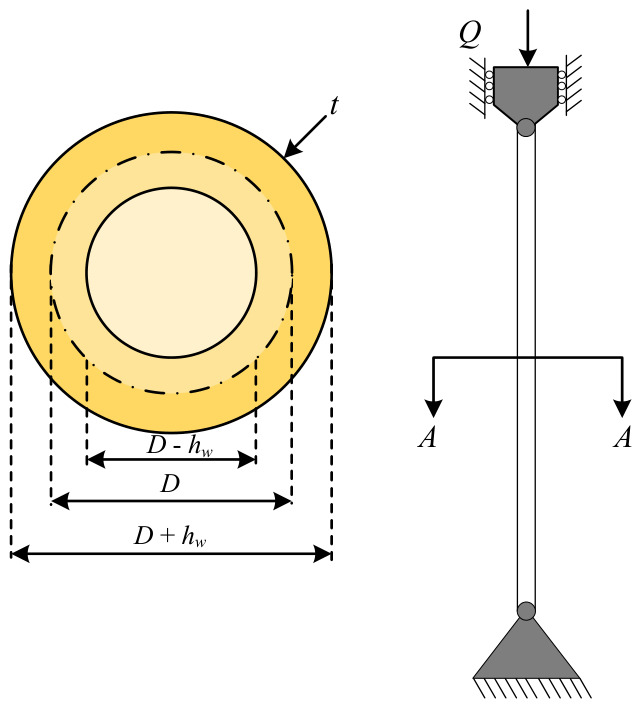
3D model of tubular column.

**Figure 21:**
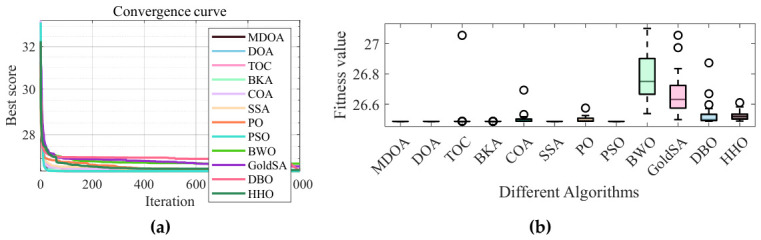
(**a**) Average convergence curves for the tubular column design problem. (**b**) Box plots for the tubular column design problem.

A 3D model of the tubular column is shown in Figure 20. Table 13 shows that MDOA achieves the optimal value of 26.48599, matching DOA, TOC, BKA, SSA, and PSO and outperforming COA, PO, GoldSA, DBO, and HHO. MDOA also achieves a mean of 26.48599 with a standard deviation of only 1.09 × 10^−14^, indicating extremely high stability, while TOC, BWO, GoldSA, and DBO show larger fluctuations. The convergence curve in Figure 21a shows that MDOA converges faster than most algorithms. The box plot in Figure 21b shows that MDOA, DOA, SSA, and PSO have highly concentrated results with almost no outliers, whereas BWO, GoldSA, and DBO exhibit poor stability.

Table 14 presents the Wilcoxon rank-sum test results. The *p*-values for MDOA and all compared algorithms are all far below 0.05, indicating highly significant differences. This statistically validates the superior performance of MDOA on this problem.

### 4.5. Moisture Content Prediction of Dendrobium Huoshanense Based on the MDOA Algorithm

The moisture content of Dendrobium huoshanense is a key indicator of its quality, storage stability, and pharmacological efficacy. Traditional oven-drying methods are accurate but cumbersome and time-consuming, making them unsuitable for real-time testing of large batches. Recent studies have explored moisture prediction using near-infrared spectroscopy combined with BP neural networks [37]. However, standard BP networks are sensitive to initial weights and thresholds, prone to local optima, and have slow convergence and poor generalization. Therefore, this paper adopts MDOA to globally optimize the initial weights and thresholds of the BP network, constructing an MDOA-BP moisture prediction model for rapid and reliable detection.

#### 4.5.1. Experimental Samples and Spectral Acquisition

**Figure 22:**
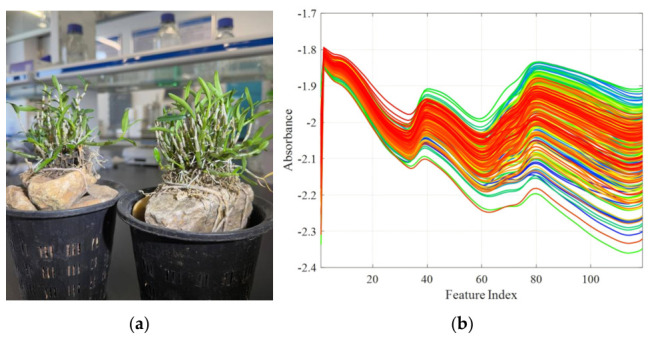
(**a**) Experimental samples of Dendrobium huoshanense. (**b**) Spectrogram for moisture content in Dendrobium huoshanense.

The experimental samples were stems of Dendrobium huoshanense. After cleaning and drying, diffuse reflectance spectra were collected using a near-infrared spectrometer at 119 wavelengths. Each sample was scanned 80 times per measurement, with the average spectrum recorded. Each sample was measured three times, and the final representative spectrum was the average of the three measurements. Figure 22a shows the experimental samples, and Figure 22b shows the near-infrared spectral curves.

#### 4.5.2. Mathematical Formulation of the Optimizer-BP Moisture Prediction Model

The near-infrared spectral data of Dendrobium huoshanense can be denoted as $X={\left[x_{1},x_{2},\ldots ,x_{N}\right]}^{T}$, where $x_{i}=\left[x_{i,1},x_{i,2},\ldots ,x_{i,p}\right]$, P=119, and $y_{i}$ is the measured moisture content. Since the moisture content is jointly affected by multiple spectral bands, the prediction task can be formulated as a nonlinear mapping, as shown in Equation (24):

(24) $$y_{i}=f(x_{i})+\varepsilon_{i}$$

where f(⋅) denotes the nonlinear relationship between spectral variables and moisture content and $\varepsilon_{i}$ is the residual error.

Before training, the spectra and moisture values are normalized to $\left[0,1\right]$, as shown in Equation (25):

(25) $${\tilde{x}}_{i,j}=\frac{x_{i,j}-x_{j}^{\min}}{x_{j}^{\max}-x_{j}^{\min}},{\tilde{y}}_{j}=\frac{y_{i}-y^{\min}}{y^{\max}-y^{\min}},$$

where $x_{j}^{\min}$ and $x_{j}^{\max}$, $y^{\min}$ and $y^{\max}$ denote the corresponding minimum and maximum values of the spectral variables and moisture content in the training set.

A three-layer BP neural network with 119 input neurons, 5 hidden neurons, and 1 output neuron is used to approximate the nonlinear mapping. Its forward propagation process is expressed as shown in Equation (26):

(26) $$\left\{\begin{array}{l} h_{i}=\phi (W_{1}{\tilde{x}}_{i}+b_{1})\\ {\hat{\tilde{y}}}_{i}=W_{2}h_{i}+b_{2} \end{array}\right.$$

where $W_{1}$, $b_{1}$, $W_{2}$, and $b_{2}$ are the weights and biases of the BP network and ϕ(⋅) is the hidden-layer activation function.

To reduce the influence of random initialization, all optimizer-based BP models optimize the same initial parameter vector $\theta =\left[\mathrm{vec}(W_{1}),b_{1},\mathrm{vec}(W_{2}),b_{2}\right]$. For the BP network used in this study, which contains 119 input neurons, 5 hidden neurons, and 1 output neuron, the optimization dimension is D=119×5+5+5×1+1=606. To ensure fair comparison, all optimizers use the same training-set mean squared error as the fitness function, as shown in Equation (27):

(27) $$F\left(\theta \right)=\frac{1}{N_{tr}}{\sum_{i=1}^{N_{tr}}\left({\tilde{y}}_{i}-{\hat{\tilde{y}}}_{i}\right)}^{2}$$

After the optimal parameter vector $\theta^{*}$ is obtained, it is assigned to the BP network as the initial weights and biases, and the normalized prediction results are transformed back to the original moisture scale by inverse normalization. The predictive performance is evaluated using $R^{2}$, RMSE, MSE, MAPE, and MAE.

#### 4.5.3. Dataset and Experimental Setup

A total of 275 valid samples were collected, each with 119 spectral features and one moisture content label. The dataset was split into training (220 samples) and test (55 samples) sets at an 8:2 ratio. The split was fixed before the experiments, and all models used the same split for fair comparison. The BP neural network has a three-layer structure: 119 input nodes, 5 hidden nodes, and 1 output node. Its initial weights and thresholds are globally optimized by MDOA with a population size of 50 and a maximum of 100 iterations. All algorithms are run independently 20 times, and the average of each metric is reported.

Evaluation metrics include the coefficient of determination ($R^{2}$), root mean square error (RMSE), mean square error (MSE), mean absolute percentage error (MAPE), and mean absolute error (MAE).

#### 4.5.4. Results and Analysis

To verify the effectiveness of MDOA-BP, it was compared with DOA-BP, TOC-BP, BKA-BP, COA-BP, SSA-BP, PSO-BP, BWO-BP, GoldSA-BP, DBO-BP, and HHO-BP. The original PO algorithm was excluded due to its extremely slow running speed (tens to hundreds of times slower than the others). All algorithms used the same settings: population size: 50, maximum iterations: 100, and 20 independent runs. Evaluation metrics included $R^{2}$, RMSE, MSE, MAPE, and MAE. The results are shown in Table 15.

As shown in Table 15, MDOA-BP outperforms all other models across all five evaluation metrics, achieving the lowest MAE, RMSE, MSE, and MAPE, as well as the highest $R^{2}$. This validates the effectiveness of MDOA in optimizing the initial weights and thresholds of the BP neural network, demonstrating superior prediction accuracy, goodness of fit, and stability. The MDOA-BP model meets the practical needs of rapid, non-destructive moisture content detection for Dendrobium huoshanense.

This section validates the proposed algorithm on five typical engineering optimization problems, ranging from classical structural optimization to near-infrared spectroscopy combined with BP neural networks. The algorithm demonstrates strong problem-solving capabilities, with significant advantages in solution quality, convergence speed, and stability. The MDOA-BP model achieves the best prediction accuracy and goodness of fit in the moisture content prediction task, further validating its potential for optimizing neural network parameters. These results collectively confirm the algorithm’s significant potential for complex constrained optimization tasks.

## 5. Summary of Work and Future Prospects

### 5.1. Summary of Work

To address the inherent defects of the Dhole Optimization Algorithm (DOA), this paper proposes MDOA, which integrates a Beta distribution-based opposition learning strategy, a DE/rand-to-best/1 differential mutation mechanism, and nonlinear parameter control. These enhancements effectively improve population diversity and balance global exploration with local exploitation.

MDOA is systematically evaluated on CEC2017 and CEC2022 (41 functions). The results show that MDOA achieves the best overall ranking, with faster convergence, higher accuracy, and stronger robustness than the other algorithms. Statistical analyses (convergence curves, box plots, Wilcoxon tests, and radar charts) confirm that MDOA rapidly approaches the global optimum with minimal fluctuations, validating its superiority over DOA and other competitors.

MDOA is applied to five engineering problems: compression spring design, speed reducer weight minimization, rolling bearing optimization, tubular column design, and moisture content prediction of Dendrobium huoshanense using near-infrared spectroscopy with a BP network. These problems span classical structural optimization to spectral data processing. The experimental results show that MDOA achieves lower means and standard deviations across all cases, demonstrating strong solution capability and stability.

In the moisture content prediction task, the MDOA-BP model achieves the lowest MAE, MAPE, MSE, and RMSE and the highest $R^{2}$, outperforming all other models. This indicates that MDOA significantly enhances the prediction accuracy and generalization of BP networks, further validating its potential in machine learning parameter optimization.

### 5.2. Future Prospects

Although the MDOA algorithm achieves strong optimization performance, further research and improvement are still needed. Future work can be pursued from the following aspects:

(1) Research on Adaptive Parameter Mechanisms.

Several parameters in this paper are still set as fixed values. Different optimization problems may require different optimal parameter combinations. In the future, adaptive parameter adjustment mechanisms or learning strategies can be introduced to enable the algorithm to dynamically adjust key parameters according to the search state, thereby further improving optimization performance.

(2) Integration with Deep Learning Models.

This paper only validates the optimization capability of MDOA on BP neural networks. In the future, MDOA can be further applied to deep neural networks, Transformer models, feature selection, and other machine learning tasks to improve the training efficiency and prediction performance of complex models.

(3) Application to More Real-World Engineering Problems.

While MDOA has been validated on five engineering optimization problems, its applicability to other complex real-world problems—such as path planning, resource scheduling, and multi-objective optimization—remains to be explored. Future work will extend MDOA to these domains to further demonstrate its practical value.

(4) Multi-Objective Extension.

The current MDOA algorithm is designed for single-objective optimization. Many real-world engineering problems involve multiple conflicting objectives. Extending MDOA to a multi-objective version and validating its performance on multi-objective benchmark functions and engineering problems is a promising research direction.

## Figures and Tables

**Table 1:** Statistical indicators for the CEC2017 test functions (F1–F10).

*f*	Results	MDOA	DOA	TOC	BKA	COA	SSA	PO	PSO	BWO	GoldSA	DBO	HHO
*F*_1_	min	1.13 × 10^4^	1.81 × 10^5^	6.54 × 10^9^	1.03 × 10^10^	1.03 × 10^11^	5.34 × 10^4^	1.61 × 10^10^	1.02 × 10^9^	9.66 × 10^10^	8.37 × 10^10^	6.06 × 10^10^	2.14 × 10^8^
	std	3.66 × 10^5^	5.64 × 10^9^	9.29 × 10^9^	2.38 × 10^10^	6.45 × 10^9^	5.04 × 10^4^	4.45 × 10^9^	4.61 × 10^9^	7.90 × 10^9^	1.13 × 10^10^	5.15 × 10^9^	9.27 × 10^7^
	avg	3.18 × 10^5^	1.27 × 10^9^	1.74 × 10^10^	2.78 × 10^10^	1.18 × 10^11^	1.16 × 10^5^	2.23 × 10^10^	6.05 × 10^9^	1.23 × 10^11^	1.03 × 10^11^	7.07 × 10^10^	3.07 × 10^8^
	rank	2	5	7	9	11	1	8	6	12	10	4	3
*F*_3_	min	5.82 × 10^4^	1.27 × 10^5^	9.19 × 10^4^	4.52 × 10^4^	1.62 × 10^5^	1.53 × 10^5^	8.97 × 10^4^	1.01 × 10^5^	1.80 × 10^5^	1.86 × 10^5^	1.57 × 10^5^	1.00 × 10^5^
	std	1.84 × 10^4^	7.52 × 10^4^	6.75 × 10^4^	2.52 × 10^4^	2.04 × 10^4^	4.68 × 10^4^	1.70 × 10^4^	3.83 × 10^4^	2.29 × 10^4^	3.20 × 10^4^	4.44 × 10^4^	2.12 × 10^4^
	avg	8.27 × 10^4^	2.43 × 10^5^	1.68 × 10^5^	7.14 × 10^4^	1.94 × 10^5^	2.28 × 10^5^	1.13 × 10^5^	1.50 × 10^5^	2.15 × 10^5^	2.35 × 10^5^	2.01 × 10^5^	1.35 × 10^5^
	rank	2	12	6	1	7	10	3	5	9	11	8	4
*F*_4_	min	4.46 × 10^2^	4.83 × 10^2^	1.59 × 10^3^	1.11 × 10^3^	3.19 × 10^4^	4.80 × 10^2^	1.21 × 10^3^	8.36 × 10^2^	3.85 × 10^4^	2.22 × 10^4^	1.17 × 10^4^	6.30 × 10^2^
	std	6.80 × 10^1^	4.50 × 10^1^	2.54 × 10^3^	1.07 × 10^3^	4.50 × 10^3^	5.08 × 10^1^	1.06 × 10^3^	5.88 × 10^2^	3.88 × 10^3^	5.05 × 10^3^	2.31 × 10^3^	8.01 × 10^1^
	avg	5.56 × 10^2^	5.80 × 10^2^	3.63 × 10^3^	2.72 × 10^3^	3.96 × 10^4^	5.82 × 10^2^	2.94 × 10^3^	1.40 × 10^3^	4.67 × 10^4^	3.13 × 10^4^	1.63 × 10^4^	8.32 × 10^2^
	rank	1	2	8	6	11	3	7	5	12	10	9	4
*F*_5_	min	6.93 × 10^2^	7.64 × 10^2^	9.30 × 10^2^	8.03 × 10^2^	1.12 × 10^3^	8.22 × 10^2^	9.28 × 10^2^	7.18 × 10^2^	1.17 × 10^3^	1.11 × 10^3^	1.02 × 10^3^	8.59 × 10^2^
	std	5.78 × 10^1^	8.90 × 10^1^	8.19 × 10^1^	8.52 × 10^1^	3.77 × 10^1^	2.61 × 10^1^	4.50 × 10^1^	5.30 × 10^1^	2.17 × 10^1^	3.54 × 10^1^	2.91 × 10^1^	2.48 × 10^1^
	avg	7.92 × 10^2^	8.72 × 10^2^	1.08 × 10^3^	8.96 × 10^2^	1.20 × 10^3^	8.83 × 10^2^	9.95 × 10^2^	8.25 × 10^2^	1.21 × 10^3^	1.18 × 10^3^	1.09 × 10^3^	9.10 × 10^2^
	rank	1	3	8	5	11	4	7	2	12	10	9	6
*F*_6_	min	6.23 × 10^2^	6.41 × 10^2^	6.63 × 10^2^	6.58 × 10^2^	6.90 × 10^2^	6.57 × 10^2^	6.63 × 10^2^	6.39 × 10^2^	6.99 × 10^2^	6.99 × 10^2^	6.77 × 10^2^	6.71 × 10^2^
	std	9.29 × 10^0^	1.32 × 10^1^	9.57 × 10^0^	7.69 × 10^0^	4.95 × 10^0^	5.03 × 10^0^	7.82 × 10^0^	7.15 × 10^0^	2.09 × 10^0^	2.93 × 10^0^	4.86 × 10^0^	3.54 × 10^0^
	avg	6.40 × 10^2^	6.62 × 10^2^	6.79 × 10^2^	6.69 × 10^2^	7.00 × 10^2^	6.66 × 10^2^	6.78 × 10^2^	6.56 × 10^2^	7.04 × 10^2^	7.04 × 10^2^	6.84 × 10^2^	6.77 × 10^2^
	rank	1	3	8	5	10	4	7	2	12	11	9	6
*F*_7_	min	1.07 × 10^3^	1.35 × 10^3^	1.58 × 10^3^	1.49 × 10^3^	1.95 × 10^3^	1.53 × 10^3^	1.46 × 10^3^	1.17 × 10^3^	2.06 × 10^3^	1.95 × 10^3^	1.70 × 10^3^	1.72 × 10^3^
	std	2.16 × 10^2^	1.34 × 10^2^	1.19 × 10^2^	9.38 × 10^1^	5.85 × 10^1^	6.52 × 10^1^	1.16 × 10^2^	1.24 × 10^2^	2.85 × 10^1^	5.32 × 10^1^	5.25 × 10^1^	7.21 × 10^1^
	avg	1.44 × 10^3^	1.59 × 10^3^	1.77 × 10^3^	1.68 × 10^3^	2.08 × 10^3^	1.74 × 10^3^	1.77 × 10^3^	1.35 × 10^3^	2.11 × 10^3^	2.07 × 10^3^	1.77 × 10^3^	1.87 × 10^3^
	rank	2	3	6	4	11	5	7	1	12	10	8	9
*F*_8_	min	1.00 × 10^3^	1.06 × 10^3^	1.24 × 10^3^	1.15 × 10^3^	1.44 × 10^3^	1.14 × 10^3^	1.22 × 10^3^	1.08 × 10^3^	1.49 × 10^3^	1.45 × 10^3^	1.26 × 10^3^	1.15 × 10^3^
	std	6.24 × 10^1^	1.06 × 10^2^	5.60 × 10^1^	7.49 × 10^1^	2.88 × 10^1^	2.90 × 10^1^	5.15 × 10^1^	5.10 × 10^1^	2.12 × 10^1^	4.27 × 10^1^	4.67 × 10^1^	2.73 × 10^1^
	avg	1.09 × 10^3^	1.25 × 10^3^	1.38 × 10^3^	1.21 × 10^3^	1.50 × 10^3^	1.21 × 10^3^	1.31 × 10^3^	1.16 × 10^3^	1.53 × 10^3^	1.51 × 10^3^	1.37 × 10^3^	1.21 × 10^3^
	rank	1	6	9	3	10	4	7	2	12	11	8	5
*F*_9_	min	6.04 × 10^3^	7.37 × 10^3^	9.62 × 10^3^	1.25 × 10^4^	3.08 × 10^4^	1.30 × 10^4^	1.38 × 10^4^	9.04 × 10^3^	3.44 × 10^4^	3.17 × 10^4^	2.56 × 10^4^	2.21 × 10^4^
	std	6.76 × 10^3^	6.08 × 10^3^	5.92 × 10^3^	4.50 × 10^3^	3.05 × 10^3^	4.34 × 10^2^	2.90 × 10^3^	1.70 × 10^3^	2.01 × 10^3^	4.01 × 10^3^	3.63 × 10^3^	3.11 × 10^3^
	avg	1.55 × 10^4^	2.16 × 10^4^	2.08 × 10^4^	1.55 × 10^4^	3.54 × 10^4^	1.34 × 10^4^	1.98 × 10^4^	1.21 × 10^4^	3.84 × 10^4^	3.73 × 10^4^	3.18 × 10^4^	2.83 × 10^4^
	rank	4	7	6	3	10	2	5	1	12	11	9	8
*F*_10_	min	6.84 × 10^3^	1.14 × 10^4^	1.20 × 10^4^	6.60 × 10^3^	1.36 × 10^4^	6.99 × 10^3^	9.74 × 10^3^	6.73 × 10^3^	1.39 × 10^4^	1.36 × 10^4^	1.12 × 10^4^	7.61 × 10^3^
	std	2.62 × 10^3^	1.07 × 10^3^	7.39 × 10^2^	1.80 × 10^3^	4.93 × 10^2^	1.14 × 10^3^	1.07 × 10^3^	6.52 × 10^2^	3.78 × 10^2^	8.18 × 10^2^	1.07 × 10^3^	9.74 × 10^2^
	avg	1.28 × 10^4^	1.44 × 10^4^	1.33 × 10^4^	8.57 × 10^3^	1.48 × 10^4^	8.89 × 10^3^	1.15 × 10^4^	7.89 × 10^3^	1.48 × 10^4^	1.51 × 10^4^	1.43 × 10^4^	9.46 × 10^3^
	rank	6	9	7	2	11	3	5	1	10	12	8	4

**Table 2:** Statistical indicators for CEC2017 hybrid functions (F11–F20).

*f*	Results	MDOA	DOA	TOC	BKA	COA	SSA	PO	PSO	BWO	GoldSA	DBO	HHO
*F*_11_	min	1.27 × 10^3^	1.31 × 10^3^	2.51 × 10^3^	1.57 × 10^3^	2.22 × 10^4^	1.30 × 10^3^	1.98 × 10^3^	1.47 × 10^3^	2.21 × 10^4^	1.65 × 10^4^	9.82 × 10^3^	1.55 × 10^3^
	std	6.09 × 10^1^	1.20 × 10^2^	7.50 × 10^3^	2.43 × 10^3^	1.85 × 10^3^	4.73 × 10^1^	1.30 × 10^3^	2.69 × 10^2^	5.88 × 10^3^	2.57 × 10^3^	2.53 × 10^3^	1.01 × 10^2^
	avg	1.39 × 10^3^	1.48 × 10^3^	7.80 × 10^3^	3.08 × 10^3^	2.73 × 10^4^	1.40 × 10^3^	3.65 × 10^3^	1.80 × 10^3^	3.38 × 10^4^	2.21 × 10^4^	1.45 × 10^4^	1.73 × 10^3^
	rank	1	3	8	6	11	2	7	5	12	10	9	4
*F*_12_	min	5.57 × 10^5^	5.19 × 10^5^	7.08 × 10^8^	4.49 × 10^7^	5.12 × 10^10^	1.76 × 10^6^	5.70 × 10^8^	1.73 × 10^7^	7.93 × 10^10^	2.40 × 10^10^	1.76 × 10^10^	5.44 × 10^7^
	std	3.48 × 10^6^	4.11 × 10^6^	2.70 × 10^9^	1.74 × 10^10^	1.61 × 10^10^	4.44 × 10^6^	2.77 × 10^9^	2.02 × 10^9^	1.45 × 10^10^	2.05 × 10^10^	8.67 × 10^9^	1.51 × 10^8^
	avg	4.34 × 10^6^	5.60 × 10^6^	3.05 × 10^9^	7.19 × 10^9^	8.82 × 10^10^	7.88 × 10^6^	3.34 × 10^9^	1.85 × 10^9^	1.06 × 10^11^	6.53 × 10^10^	3.55 × 10^10^	2.19 × 10^8^
	rank	1	2	6	8	11	3	7	5	12	10	9	4
*F*_13_	min	2.36 × 10^3^	3.82 × 10^3^	3.40 × 10^7^	8.18 × 10^5^	2.37 × 10^10^	7.73 × 10^3^	1.56 × 10^6^	3.75 × 10^4^	2.99 × 10^10^	1.69 × 10^10^	4.61 × 10^9^	1.80 × 10^6^
	std	6.33 × 10^3^	1.09 × 10^4^	9.38 × 10^8^	2.05 × 10^9^	1.51 × 10^10^	1.27 × 10^4^	1.53 × 10^8^	2.35 × 10^8^	1.34 × 10^10^	1.26 × 10^10^	4.45 × 10^9^	1.32 × 10^6^
	avg	9.19 × 10^3^	1.28 × 10^4^	6.45 × 10^8^	6.53 × 10^8^	5.15 × 10^10^	2.22 × 10^4^	1.18 × 10^8^	1.22 × 10^8^	6.47 × 10^10^	3.59 × 10^10^	1.40 × 10^10^	4.34 × 10^6^
	rank	1	2	7	8	11	3	5	6	12	10	9	4
*F*_14_	min	2.85 × 10^3^	1.40 × 10^4^	3.79 × 10^4^	3.52 × 10^4^	5.30 × 10^6^	6.56 × 10^4^	2.65 × 10^5^	1.79 × 10^4^	4.50 × 10^7^	1.10 × 10^7^	1.30 × 10^6^	4.13 × 10^5^
	std	6.76 × 10^4^	8.11 × 10^4^	1.85 × 10^6^	6.30 × 10^6^	6.54 × 10^7^	1.83 × 10^5^	1.60 × 10^6^	4.03 × 10^5^	1.30 × 10^8^	4.70 × 10^7^	5.74 × 10^6^	1.78 × 10^6^
	avg	7.56 × 10^4^	1.34 × 10^5^	1.43 × 10^6^	2.38 × 10^6^	7.67 × 10^7^	3.31 × 10^5^	2.03 × 10^6^	2.30 × 10^5^	2.47 × 10^8^	7.18 × 10^7^	7.50 × 10^6^	2.41 × 10^6^
	rank	1	2	6	8	11	4	5	3	12	10	7	9
*F*_15_	min	1.91 × 10^3^	3.09 × 10^3^	3.31 × 10^5^	4.18 × 10^4^	5.00 × 10^9^	3.12 × 10^3^	9.64 × 10^4^	7.48 × 10^3^	6.30 × 10^9^	2.30 × 10^9^	1.43 × 10^8^	2.90 × 10^5^
	std	6.85 × 10^3^	6.23 × 10^3^	6.90 × 10^7^	2.71 × 10^8^	2.52 × 10^9^	7.77 × 10^3^	2.01 × 10^7^	2.19 × 10^7^	3.37 × 10^9^	3.17 × 10^9^	9.33 × 10^8^	3.91 × 10^5^
	avg	9.22 × 10^3^	1.36 × 10^4^	4.20 × 10^7^	6.10 × 10^7^	9.08 × 10^9^	1.74 × 10^4^	1.01 × 10^7^	7.15 × 10^6^	1.55 × 10^10^	6.87 × 10^9^	1.63 × 10^9^	8.03 × 10^5^
	rank	1	2	8	9	11	3	6	5	12	10	7	4
*F*_16_	min	2.50 × 10^3^	3.01 × 10^3^	4.04 × 10^3^	3.27 × 10^3^	7.56 × 10^3^	3.03 × 10^3^	4.08 × 10^3^	3.02 × 10^3^	9.15 × 10^3^	6.41 × 10^3^	4.89 × 10^3^	3.46 × 10^3^
	std	4.32 × 10^2^	5.74 × 10^2^	9.67 × 10^2^	4.80 × 10^2^	1.71 × 10^3^	4.89 × 10^2^	4.95 × 10^2^	4.40 × 10^2^	2.02 × 10^3^	1.93 × 10^3^	3.38 × 10^2^	5.31 × 10^2^
	avg	3.25 × 10^3^	3.97 × 10^3^	5.15 × 10^3^	3.99 × 10^3^	9.77 × 10^3^	3.75 × 10^3^	5.13 × 10^3^	3.75 × 10^3^	1.27 × 10^4^	9.47 × 10^3^	5.48 × 10^3^	4.35 × 10^3^
	rank	1	4	8	5	11	3	7	2	12	10	9	6
*F*_17_	min	2.75 × 10^3^	2.67 × 10^3^	3.39 × 10^3^	3.09 × 10^3^	4.90 × 10^3^	2.97 × 10^3^	3.50 × 10^3^	2.88 × 10^3^	5.75 × 10^3^	4.80 × 10^3^	4.08 × 10^3^	2.78 × 10^3^
	std	2.78 × 10^2^	4.52 × 10^2^	4.49 × 10^2^	3.43 × 10^2^	1.05 × 10^4^	3.83 × 10^2^	4.69 × 10^2^	3.78 × 10^2^	2.12 × 10^4^	5.10 × 10^3^	5.92 × 10^2^	3.52 × 10^2^
	avg	3.25 × 10^3^	3.42 × 10^3^	4.18 × 10^3^	3.52 × 10^3^	1.29 × 10^4^	3.60 × 10^3^	4.23 × 10^3^	3.43 × 10^3^	2.99 × 10^4^	9.20 × 10^3^	4.83 × 10^3^	3.64 × 10^3^
	rank	1	2	7	4	11	5	8	3	12	10	9	6
*F*_18_	min	1.25 × 10^5^	7.07 × 10^4^	2.38 × 10^5^	1.43 × 10^5^	5.54 × 10^7^	3.38 × 10^5^	1.30 × 10^6^	2.92 × 10^5^	1.76 × 10^8^	1.11 × 10^7^	3.76 × 10^6^	9.50 × 10^5^
	std	5.51 × 10^5^	3.30 × 10^5^	1.89 × 10^7^	2.10 × 10^7^	8.36 × 10^7^	1.51 × 10^6^	1.40 × 10^7^	3.19 × 10^6^	1.74 × 10^8^	1.39 × 10^8^	1.60 × 10^7^	3.41 × 10^6^
	avg	6.22 × 10^5^	5.06 × 10^5^	9.08 × 10^6^	8.14 × 10^6^	1.73 × 10^8^	2.26 × 10^6^	1.42 × 10^7^	2.20 × 10^6^	3.51 × 10^8^	2.59 × 10^8^	1.76 × 10^7^	5.71 × 10^6^
	rank	2	1	7	6	10	4	8	3	12	11	9	5
*F*_19_	min	2.19 × 10^3^	2.27 × 10^3^	6.90 × 10^5^	9.16 × 10^4^	1.62 × 10^9^	2.27 × 10^3^	4.07 × 10^5^	6.16 × 10^3^	1.40 × 10^9^	2.55 × 10^8^	1.79 × 10^8^	1.16 × 10^5^
	std	1.26 × 10^4^	1.33 × 10^4^	3.14 × 10^8^	8.76 × 10^7^	1.87 × 10^9^	1.52 × 10^4^	1.25 × 10^7^	7.85 × 10^4^	1.79 × 10^9^	1.30 × 10^9^	9.91 × 10^8^	1.22 × 10^6^
	avg	1.42 × 10^4^	1.55 × 10^4^	8.74 × 10^7^	3.52 × 10^7^	4.57 × 10^9^	2.38 × 10^4^	9.42 × 10^6^	4.67 × 10^4^	6.59 × 10^9^	2.23 × 10^9^	1.38 × 10^9^	1.67 × 10^6^
	rank	1	2	8	7	11	3	6	4	12	10	9	5
*F*_20_	min	2.65 × 10^3^	3.06 × 10^3^	3.06 × 10^3^	2.79 × 10^3^	3.83 × 10^3^	3.26 × 10^3^	3.17 × 10^3^	2.79 × 10^3^	3.81 × 10^3^	3.68 × 10^3^	2.97 × 10^3^	3.04 × 10^3^
	std	2.53 × 10^2^	3.83 × 10^2^	3.35 × 10^2^	2.92 × 10^2^	2.19 × 10^2^	2.11 × 10^2^	2.27 × 10^2^	3.51 × 10^2^	1.90 × 10^2^	3.01 × 10^2^	3.61 × 10^2^	2.58 × 10^2^
	avg	3.18 × 10^3^	3.59 × 10^3^	3.86 × 10^3^	3.24 × 10^3^	4.19 × 10^3^	3.70 × 10^3^	3.64 × 10^3^	3.32 × 10^3^	4.13 × 10^3^	4.31 × 10^3^	3.75 × 10^3^	3.55 × 10^3^
	rank	1	5	9	2	11	7	6	3	10	12	8	4

**Table 3:** Statistical indicators for CEC2017 composite functions (F21–F30).

f	Results	MDOA	DOA	TOC	BKA	COA	SSA	PO	PSO	BWO	GoldSA	DBO	HHO
*F*_21_	min	2.48 × 10^3^	2.56 × 10^3^	2.79 × 10^3^	2.70 × 10^3^	3.14 × 10^3^	2.68 × 10^3^	2.70 × 10^3^	2.55 × 10^3^	3.25 × 10^3^	3.07 × 10^3^	2.87 × 10^3^	2.76 × 10^3^
	std	4.18 × 10^1^	1.16 × 10^2^	9.04 × 10^1^	1.36 × 10^2^	9.53 × 10^1^	9.49 × 10^1^	6.09 × 10^1^	7.84 × 10^1^	6.14 × 10^1^	8.82 × 10^1^	3.98 × 10^1^	7.12 × 10^1^
	avg	2.53 × 10^3^	2.74 × 10^3^	2.90 × 10^3^	2.85 × 10^3^	3.29 × 10^3^	2.83 × 10^3^	2.82 × 10^3^	2.67 × 10^3^	3.37 × 10^3^	3.21 × 10^3^	2.96 × 10^3^	2.89 × 10^3^
	rank	1	3	8	6	11	5	4	2	12	10	9	7
*F*_22_	min	8.58 × 10^3^	2.32 × 10^3^	1.21 × 10^4^	9.19 × 10^3^	1.53 × 10^4^	8.27 × 10^3^	1.17 × 10^4^	3.37 × 10^3^	1.57 × 10^4^	1.55 × 10^4^	1.35 × 10^4^	9.85 × 10^3^
	std	2.86 × 10^3^	4.28 × 10^3^	8.83 × 10^2^	2.44 × 10^3^	6.80 × 10^2^	9.25 × 10^2^	9.39 × 10^2^	1.90 × 10^3^	4.79 × 10^2^	6.38 × 10^2^	1.14 × 10^3^	8.13 × 10^2^
	avg	1.26 × 10^4^	1.47 × 10^4^	1.45 × 10^4^	1.21 × 10^4^	1.69 × 10^4^	1.01 × 10^4^	1.32 × 10^4^	9.79 × 10^3^	1.68 × 10^4^	1.66 × 10^4^	1.58 × 10^4^	1.17 × 10^4^
	rank	5	8	7	4	12	2	6	1	11	10	9	3
*F*_23_	min	2.91 × 10^3^	3.02 × 10^3^	3.56 × 10^3^	3.45 × 10^3^	4.14 × 10^3^	3.29 × 10^3^	3.28 × 10^3^	3.29 × 10^3^	4.46 × 10^3^	3.95 × 10^3^	3.51 × 10^3^	3.49 × 10^3^
	std	1.11 × 10^2^	1.57 × 10^2^	1.93 × 10^2^	3.11 × 10^2^	1.82 × 10^2^	1.02 × 10^2^	1.47 × 10^2^	1.25 × 10^2^	1.81 × 10^2^	2.77 × 10^2^	1.29 × 10^2^	2.02 × 10^2^
	avg	3.13 × 10^3^	3.25 × 10^3^	3.89 × 10^3^	3.88 × 10^3^	4.50 × 10^3^	3.48 × 10^3^	3.52 × 10^3^	3.50 × 10^3^	4.87 × 10^3^	4.50 × 10^3^	3.77 × 10^3^	3.91 × 10^3^
	rank	1	2	8	7	10	3	5	4	12	11	6	9
*F*_24_	min	3.14 × 10^3^	3.16 × 10^3^	3.66 × 10^3^	3.55 × 10^3^	4.49 × 10^3^	3.39 × 10^3^	3.44 × 10^3^	3.41 × 10^3^	5.17 × 10^3^	4.29 × 10^3^	3.72 × 10^3^	3.89 × 10^3^
	std	1.62 × 10^2^	1.57 × 10^2^	3.21 × 10^2^	2.13 × 10^2^	2.44 × 10^2^	1.76 × 10^2^	1.25 × 10^2^	1.46 × 10^2^	3.43 × 10^2^	5.46 × 10^2^	2.14 × 10^2^	2.25 × 10^2^
	avg	3.31 × 10^3^	3.43 × 10^3^	4.07 × 10^3^	3.85 × 10^3^	4.88 × 10^3^	3.61 × 10^3^	3.66 × 10^3^	3.64 × 10^3^	5.98 × 10^3^	4.79 × 10^3^	4.13 × 10^3^	4.26 × 10^3^
	rank	1	2	8	6	11	3	6	4	12	10	7	9
*F*_25_	min	3.02 × 10^3^	3.03 × 10^3^	4.28 × 10^3^	3.42 × 10^3^	1.38 × 10^4^	3.02 × 10^3^	3.88 × 10^3^	3.30 × 10^3^	1.52 × 10^4^	1.09 × 10^4^	9.13 × 10^3^	3.17 × 10^3^
	std	3.55 × 10^1^	3.26 × 10^1^	8.12 × 10^2^	1.86 × 10^3^	1.18 × 10^3^	3.38 × 10^1^	3.91 × 10^2^	2.28 × 10^2^	9.95 × 10^2^	1.56 × 10^3^	8.87 × 10^2^	5.22 × 10^1^
	avg	3.09 × 10^3^	3.10 × 10^3^	5.22 × 10^3^	4.67 × 10^3^	1.59 × 10^4^	3.10 × 10^3^	4.49 × 10^3^	3.64 × 10^3^	1.71 × 10^4^	1.33 × 10^4^	1.10 × 10^4^	3.27 × 10^3^
	rank	1	2	8	7	11	3	6	4	12	10	9	5
*F*_26_	min	3.07 × 10^3^	3.14 × 10^3^	9.53 × 10^3^	9.04 × 10^3^	1.56 × 10^4^	3.12 × 10^3^	8.04 × 10^3^	5.24 × 10^3^	1.73 × 10^4^	1.65 × 10^4^	1.20 × 10^4^	5.22 × 10^3^
	std	3.58 × 10^3^	3.93 × 10^3^	2.62 × 10^3^	1.95 × 10^3^	8.26 × 10^2^	2.82 × 10^3^	1.56 × 10^3^	1.69 × 10^3^	5.83 × 10^2^	7.66 × 10^2^	8.33 × 10^2^	1.77 × 10^3^
	avg	7.81 × 10^3^	7.66 × 10^3^	1.42 × 10^4^	1.27 × 10^4^	1.75 × 10^4^	9.17 × 10^3^	1.27 × 10^4^	1.05 × 10^4^	1.87 × 10^4^	1.78 × 10^4^	1.36 × 10^4^	1.08 × 10^4^
	rank	2	1	9	6	10	3	7	4	12	11	8	5
*F*_27_	min	3.41 × 10^3^	3.35 × 10^3^	3.74 × 10^3^	3.70 × 10^3^	5.58 × 10^3^	3.43 × 10^3^	3.73 × 10^3^	3.60 × 10^3^	5.63 × 10^3^	5.28 × 10^3^	4.29 × 10^3^	3.99 × 10^3^
	std	1.76 × 10^2^	1.90 × 10^2^	3.21 × 10^2^	3.89 × 10^2^	7.13 × 10^2^	1.98 × 10^2^	2.32 × 10^2^	1.79 × 10^2^	1.13 × 10^3^	7.84 × 10^2^	3.38 × 10^2^	5.78 × 10^2^
	avg	3.67 × 10^3^	3.71 × 10^3^	4.20 × 10^3^	4.25 × 10^3^	6.83 × 10^3^	3.68 × 10^3^	4.03 × 10^3^	3.85 × 10^3^	7.76 × 10^3^	7.00 × 10^3^	4.92 × 10^3^	4.72 × 10^3^
	rank	1	3	6	7	10	2	5	4	12	11	9	8
*F*_28_	min	3.31 × 10^3^	3.29 × 10^3^	3.92 × 10^3^	4.18 × 10^3^	1.16 × 10^4^	3.32 × 10^3^	4.80 × 10^3^	3.89 × 10^3^	1.28 × 10^4^	1.03 × 10^4^	7.22 × 10^3^	3.63 × 10^3^
	std	3.70 × 10^1^	4.60 × 10^1^	7.68 × 10^2^	1.62 × 10^3^	1.40 × 10^3^	3.73 × 10^1^	4.50 × 10^2^	7.65 × 10^2^	1.68 × 10^3^	1.25 × 10^3^	7.13 × 10^2^	1.23 × 10^2^
	avg	3.35 × 10^3^	3.37 × 10^3^	5.49 × 10^3^	5.41 × 10^3^	1.40 × 10^4^	3.38 × 10^3^	5.41 × 10^3^	4.73 × 10^3^	1.67 × 10^4^	1.24 × 10^4^	8.23 × 10^3^	3.82 × 10^3^
	rank	1	2	8	7	11	3	6	5	12	10	9	4
*F*_29_	min	4.25 × 10^3^	4.15 × 10^3^	5.62 × 10^3^	5.09 × 10^3^	1.60 × 10^4^	4.25 × 10^3^	5.62 × 10^3^	4.84 × 10^3^	8.80 × 10^4^	1.32 × 10^4^	7.73 × 10^3^	5.39 × 10^3^
	std	3.33 × 10^2^	4.74 × 10^2^	1.31 × 10^3^	5.03 × 10^3^	9.78 × 10^4^	4.66 × 10^2^	8.99 × 10^2^	5.34 × 10^2^	3.61 × 10^5^	6.04 × 10^4^	2.06 × 10^3^	6.99 × 10^2^
	avg	4.85 × 10^3^	4.83 × 10^3^	8.09 × 10^3^	8.21 × 10^3^	1.01 × 10^5^	5.07 × 10^3^	7.25 × 10^3^	5.55 × 10^3^	4.99 × 10^5^	6.55 × 10^4^	1.10 × 10^4^	6.33 × 10^3^
	rank	2	1	7	8	11	3	6	4	12	10	9	5
*F*_30_	min	9.21 × 10^5^	8.89 × 10^5^	3.32 × 10^7^	1.79 × 10^7^	2.78 × 10^9^	6.86 × 10^5^	1.27 × 10^8^	7.74 × 10^6^	8.67 × 10^9^	1.52 × 10^9^	5.76 × 10^8^	3.09 × 10^7^
	std	4.41 × 10^5^	9.62 × 10^5^	3.76 × 10^8^	1.12 × 10^9^	3.19 × 10^9^	4.62 × 10^5^	6.60 × 10^7^	9.61 × 10^6^	3.05 × 10^9^	2.04 × 10^9^	8.82 × 10^8^	1.82 × 10^7^
	avg	1.40 × 10^6^	1.72 × 10^6^	2.90 × 10^8^	6.53 × 10^8^	8.73 × 10^9^	1.36 × 10^6^	2.06 × 10^8^	2.37 × 10^7^	1.38 × 10^10^	4.44 × 10^9^	1.62 × 10^9^	5.67 × 10^7^
	rank	2	3	7	8	11	1	6	4	12	10	9	5

**Table 4:** Wilcoxon rank-sum test *p*-values for MDOA and other algorithms on CEC2017 functions.

	DOA	TOC	BKA	COA	SSA	PO	PSO	BWO	GoldSA	DBO	HHO
*F*_1_	1.05 × 10^−6^	6.80 × 10^−8^	6.80 × 10^−8^	6.80 × 10^−8^	2.39 × 10^−1^	6.80 × 10^−8^	6.80 × 10^−8^	6.80 × 10^−8^	6.80 × 10^−8^	6.80 × 10^−8^	6.80 × 10^−8^
*F*_3_	6.80 × 10^−8^	4.54 × 10^−7^	2.39 × 10^−2^	6.80 × 10^−8^	6.80 × 10^−8^	5.90 × 10^−5^	9.13 × 10^−7^	6.80 × 10^−8^	6.80 × 10^−8^	6.80 × 10^−8^	7.95 × 10^−7^
*F*_4_	9.62 × 10^−2^	6.80 × 10^−8^	6.80 × 10^−8^	6.80 × 10^−8^	6.01 × 10^−2^	6.80 × 10^−8^	6.80 × 10^−8^	6.80 × 10^−8^	6.80 × 10^−8^	6.80 × 10^−8^	1.43 × 10^−7^
*F*_5_	2.56 × 10^−3^	6.80 × 10^−8^	5.90 × 10^−5^	6.80 × 10^−8^	3.07 × 10^−6^	6.80 × 10^−8^	1.02 × 10^−1^	6.80 × 10^−8^	6.80 × 10^−8^	6.80 × 10^−8^	2.22 × 10^−7^
*F*_6_	4.54 × 10^−6^	9.17 × 10^−8^	2.56 × 10^−7^	6.80 × 10^−8^	5.23 × 10^−7^	9.17 × 10^−8^	5.87 × 10^−6^	6.80 × 10^−8^	6.80 × 10^−8^	6.80 × 10^−8^	6.80 × 10^−8^
*F*_7_	2.75 × 10^−2^	9.75 × 10^−6^	2.75 × 10^−4^	6.80 × 10^−8^	1.81 × 10^−5^	1.10 × 10^−5^	2.62 × 10^−1^	6.80 × 10^−8^	6.80 × 10^−8^	6.67 × 10^−6^	1.92 × 10^−7^
*F*_8_	9.75 × 10^−6^	6.80 × 10^−8^	2.04 × 10^−5^	6.80 × 10^−8^	1.80 × 10^−6^	6.80 × 10^−8^	1.35 × 10^−3^	6.80 × 10^−8^	6.80 × 10^−8^	6.80 × 10^−8^	2.36 × 10^−6^
*F*_9_	8.35 × 10^−3^	1.79 × 10^−2^	9.89 × 10^−1^	6.80 × 10^−8^	2.98 × 10^−1^	1.55 × 10^−2^	9.09 × 10^−2^	6.80 × 10^−8^	6.80 × 10^−8^	2.56 × 10^−7^	1.80 × 10^−6^
*F*_10_	2.39 × 10^−2^	3.10 × 10^−1^	2.22 × 10^−4^	2.75 × 10^−4^	1.16 × 10^−4^	3.97 × 10^−3^	1.10 × 10^−5^	8.29 × 10^−5^	1.04 × 10^−4^	4.68 × 10^−2^	4.16 × 10^−4^
*F*_11_	1.23 × 10^−2^	6.80 × 10^−8^	6.80 × 10^−8^	6.80 × 10^−8^	5.98 × 10^−1^	6.80 × 10^−8^	9.17 × 10^−8^	6.80 × 10^−8^	6.80 × 10^−8^	6.80 × 10^−8^	6.80 × 10^−8^
*F*_12_	3.10 × 10^−1^	6.80 × 10^−8^	6.80 × 10^−8^	6.80 × 10^−8^	8.35 × 10^−3^	6.80 × 10^−8^	6.80 × 10^−8^	6.80 × 10^−8^	6.80 × 10^−8^	6.80 × 10^−8^	6.80 × 10^−8^
*F*_13_	2.39 × 10^−1^	6.80 × 10^−8^	6.80 × 10^−8^	6.80 × 10^−8^	1.16 × 10^−4^	6.80 × 10^−8^	6.80 × 10^−8^	6.80 × 10^−8^	6.80 × 10^−8^	6.80 × 10^−8^	6.80 × 10^−8^
*F*_14_	1.14 × 10^−2^	1.41 × 10^−5^	2.23 × 10^−2^	6.80 × 10^−8^	3.07 × 10^−6^	6.80 × 10^−8^	1.08 × 10^−1^	6.80 × 10^−8^	6.80 × 10^−8^	6.80 × 10^−8^	6.80 × 10^−8^
*F*_15_	4.39 × 10^−2^	6.80 × 10^−8^	6.80 × 10^−8^	6.80 × 10^−8^	8.36 × 10^−4^	6.80 × 10^−8^	1.25 × 10^−5^	6.80 × 10^−8^	6.80 × 10^−8^	6.80 × 10^−8^	6.80 × 10^−8^
*F*_16_	1.04 × 10^−4^	7.90 × 10^−8^	6.61 × 10^−5^	6.80 × 10^−8^	3.06 × 10^−3^	6.80 × 10^−8^	1.63 × 10^−3^	6.80 × 10^−8^	6.80 × 10^−8^	6.80 × 10^−8^	6.92 × 10^−7^
*F*_17_	2.73 × 10^−1^	7.95 × 10^−7^	9.05 × 10^−3^	6.80 × 10^−8^	3.64 × 10^−3^	1.23 × 10^−7^	1.81 × 10^−1^	6.80 × 10^−8^	6.80 × 10^−8^	6.80 × 10^−8^	5.09 × 10^−4^
*F*_18_	9.03 × 10^−1^	1.81 × 10^−5^	5.31 × 10^−2^	6.80 × 10^−8^	2.92 × 10^−5^	1.23 × 10^−7^	2.07 × 10^−2^	6.80 × 10^−8^	6.80 × 10^−8^	6.80 × 10^−8^	1.66 × 10^−7^
*F*_19_	7.15 × 10^−1^	6.80 × 10^−8^	6.80 × 10^−8^	6.80 × 10^−8^	3.85 × 10^−2^	6.80 × 10^−8^	1.55 × 10^−2^	6.80 × 10^−8^	6.80 × 10^−8^	6.80 × 10^−8^	6.80 × 10^−8^
*F*_20_	3.75 × 10^−4^	1.80 × 10^−6^	7.15 × 10^−1^	6.80 × 10^−8^	1.38 × 10^−6^	5.17 × 10^−6^	2.18 × 10^−1^	6.80 × 10^−8^	6.80 × 10^−8^	8.60 × 10^−6^	1.79 × 10^−4^
*F*_21_	2.96 × 10^−7^	6.80 × 10^−8^	6.80 × 10^−8^	6.80 × 10^−8^	6.80 × 10^−8^	6.80 × 10^−8^	3.94 × 10^−7^	6.80 × 10^−8^	6.80 × 10^−8^	6.80 × 10^−8^	6.80 × 10^−8^
*F*_22_	1.12 × 10^−3^	1.56 × 10^−1^	9.89 × 10^−1^	3.94 × 10^−7^	3.15 × 10^−2^	9.03 × 10^−1^	9.79 × 10^−3^	1.43 × 10^−7^	3.42 × 10^−7^	6.61 × 10^−5^	4.25 × 10^−1^
*F*_23_	1.06 × 10^−2^	6.80 × 10^−8^	6.80 × 10^−8^	6.80 × 10^−8^	1.23 × 10^−7^	1.66 × 10^−7^	1.43 × 10^−7^	6.80 × 10^−8^	6.80 × 10^−8^	6.80 × 10^−8^	6.80 × 10^−8^
*F*_24_	9.05 × 10^−3^	1.43 × 10^−7^	5.23 × 10^−7^	6.80 × 10^−8^	4.54 × 10^−6^	3.07 × 10^−6^	4.54 × 10^−6^	6.80 × 10^−8^	6.80 × 10^−8^	7.90 × 10^−8^	6.80 × 10^−8^
*F*_25_	9.89 × 10^−1^	6.80 × 10^−8^	6.80 × 10^−8^	6.80 × 10^−8^	5.98 × 10^−1^	6.80 × 10^−8^	6.80 × 10^−8^	6.80 × 10^−8^	6.80 × 10^−8^	6.80 × 10^−8^	6.80 × 10^−8^
*F*_26_	9.03 × 10^−1^	7.95 × 10^−7^	1.41 × 10^−5^	6.80 × 10^−8^	2.73 × 10^−1^	5.17 × 10^−6^	2.56 × 10^−2^	6.80 × 10^−8^	6.80 × 10^−8^	6.80 × 10^−8^	5.56 × 10^−3^
*F*_27_	5.43 × 10^−1^	1.05 × 10^−6^	1.58 × 10^−6^	6.80 × 10^−8^	7.35 × 10^−1^	6.67 × 10^−6^	8.35 × 10^−3^	6.80 × 10^−8^	6.80 × 10^−8^	6.80 × 10^−8^	1.43 × 10^−7^
*F*_28_	2.50 × 10^−1^	6.80 × 10^−8^	6.80 × 10^−8^	6.80 × 10^−8^	4.11 × 10^−2^	6.80 × 10^−8^	6.80 × 10^−8^	6.80 × 10^−8^	6.80 × 10^−8^	6.80 × 10^−8^	6.80 × 10^−8^
*F*_29_	3.94 × 10^−1^	6.80 × 10^−8^	1.43 × 10^−7^	6.80 × 10^−8^	1.02 × 10^−1^	6.80 × 10^−8^	1.60 × 10^−5^	6.80 × 10^−8^	6.80 × 10^−8^	6.80 × 10^−8^	9.17 × 10^−8^
*F*_30_	4.57 × 10^−1^	6.80 × 10^−8^	6.80 × 10^−8^	6.80 × 10^−8^	7.35 × 10^−1^	6.80 × 10^−8^	6.80 × 10^−8^	6.80 × 10^−8^	6.80 × 10^−8^	6.80 × 10^−8^	6.80 × 10^−8^

**Table 5:** Statistical indicators for CEC2022.

*f*	Results	MDOA	DOA	TOC	BKA	COA	SSA	PO	PSO	BWO	GoldSA	DBO	HHO
*F*_1_	min	3.01 × 10^2^	1.32 × 10^3^	1.50 × 10^3^	4.48 × 10^2^	3.24 × 10^4^	1.71 × 10^3^	2.99 × 10^3^	3.33 × 10^2^	4.89 × 10^4^	3.62 × 10^4^	2.09 × 10^4^	1.14 × 10^3^
	std	9.88 × 10^2^	6.40 × 10^3^	1.76 × 10^4^	1.49 × 10^3^	1.29 × 10^4^	3.61 × 10^3^	3.31 × 10^3^	1.95 × 10^3^	8.70 × 10^7^	3.39 × 10^4^	5.19 × 10^3^	2.75 × 10^3^
	avg	9.72 × 10^2^	6.30 × 10^3^	1.97 × 10^4^	1.89 × 10^3^	4.83 × 10^4^	7.55 × 10^3^	7.98 × 10^3^	1.79 × 10^3^	2.86 × 10^7^	9.56 × 10^4^	2.84 × 10^4^	8.19 × 10^3^
	rank	1	4	8	3	10	5	6	2	12	11	9	7
*F*_2_	min	4.03 × 10^2^	4.00 × 10^2^	4.55 × 10^2^	4.48 × 10^2^	1.45 × 10^3^	4.00 × 10^2^	4.71 × 10^2^	4.07 × 10^2^	2.44 × 10^3^	1.24 × 10^3^	7.73 × 10^2^	4.50 × 10^2^
	std	1.96 × 10^1^	1.74 × 10^1^	7.54 × 10^1^	4.75 × 10^2^	6.30 × 10^2^	2.18 × 10^1^	8.18 × 10^1^	5.41 × 10^1^	8.99 × 10^2^	6.58 × 10^2^	9.62 × 10^1^	1.77 × 10^1^
	avg	4.44 × 10^2^	4.55 × 10^2^	5.46 × 10^2^	6.99 × 10^2^	2.89 × 10^3^	4.52 × 10^2^	5.52 × 10^2^	4.76 × 10^2^	4.54 × 10^3^	2.28 × 10^3^	8.95 × 10^2^	4.72 × 10^2^
	rank	1	3	6	8	11	2	7	5	12	10	9	4
*F*_3_	min	6.01 × 10^2^	6.01 × 10^2^	6.22 × 10^2^	6.32 × 10^2^	6.61 × 10^2^	6.19 × 10^2^	6.34 × 10^2^	6.10 × 10^2^	6.72 × 10^2^	6.57 × 10^2^	6.41 × 10^2^	6.35 × 10^2^
	std	1.01 × 10^1^	1.11 × 10^1^	1.26 × 10^1^	8.13 × 10^0^	7.65 × 10^0^	8.65 × 10^0^	1.11 × 10^1^	1.25 × 10^1^	7.03 × 10^0^	9.52 × 10^0^	6.76 × 10^0^	1.11 × 10^1^
	avg	6.12 × 10^2^	6.12 × 10^2^	6.45 × 10^2^	6.54 × 10^2^	6.75 × 10^2^	6.33 × 10^2^	6.52 × 10^2^	6.32 × 10^2^	6.87 × 10^2^	6.80 × 10^2^	6.54 × 10^2^	6.60 × 10^2^
	rank	1	2	5	8	10	4	6	3	12	11	7	9
*F*_4_	min	8.31 × 10^2^	8.26 × 10^2^	8.54 × 10^2^	8.39 × 10^2^	9.34 × 10^2^	8.75 × 10^2^	8.50 × 10^2^	8.35 × 10^2^	9.59 × 10^2^	9.37 × 10^2^	9.07 × 10^2^	8.62 × 10^2^
	std	2.25 × 10^1^	3.36 × 10^1^	2.13 × 10^1^	1.91 × 10^1^	2.16 × 10^1^	1.18 × 10^1^	2.07 × 10^1^	1.65 × 10^1^	8.78 × 10^0^	1.67 × 10^1^	1.64 × 10^1^	1.24 × 10^1^
	avg	8.62 × 10^2^	8.98 × 10^2^	9.02 × 10^2^	8.83 × 10^2^	9.72 × 10^2^	8.92 × 10^2^	8.94 × 10^2^	8.62 × 10^2^	9.74 × 10^2^	9.75 × 10^2^	9.33 × 10^2^	8.87 × 10^2^
	rank	2	7	8	3	10	5	6	1	11	12	9	4
*F*_5_	min	9.17 × 10^2^	9.72 × 10^2^	1.04 × 10^3^	1.39 × 10^3^	2.69 × 10^3^	1.86 × 10^3^	1.79 × 10^3^	9.17 × 10^2^	3.20 × 10^3^	3.07 × 10^3^	2.10 × 10^3^	2.37 × 10^3^
	std	3.55 × 10^2^	5.64 × 10^2^	3.83 × 10^2^	3.70 × 10^2^	3.88 × 10^2^	1.69 × 10^2^	2.75 × 10^2^	5.73 × 10^2^	3.12 × 10^2^	3.94 × 10^2^	3.21 × 10^2^	2.48 × 10^2^
	avg	1.29 × 10^3^	1.56 × 10^3^	1.65 × 10^3^	2.12 × 10^3^	3.39 × 10^3^	2.42 × 10^3^	2.37 × 10^3^	1.77 × 10^3^	3.82 × 10^3^	3.80 × 10^3^	2.58 × 10^3^	2.82 × 10^3^
	rank	1	2	3	5	10	7	6	4	12	11	8	9
*F*_6_	min	1.90 × 10^3^	1.89 × 10^3^	5.30 × 10^3^	3.64 × 10^3^	9.42 × 10^8^	1.87 × 10^3^	3.14 × 10^3^	2.04 × 10^3^	1.54 × 10^9^	2.00 × 10^8^	1.44 × 10^6^	3.59 × 10^4^
	std	5.32 × 10^3^	6.87 × 10^3^	1.46 × 10^6^	4.50 × 10^7^	7.26 × 10^8^	5.92 × 10^3^	7.88 × 10^5^	4.32 × 10^3^	1.52 × 10^9^	1.60 × 10^9^	2.48 × 10^7^	6.53 × 10^4^
	avg	6.67 × 10^3^	8.73 × 10^3^	6.71 × 10^5^	1.01 × 10^7^	2.27 × 10^9^	4.99 × 10^3^	5.40 × 10^5^	4.91 × 10^3^	4.24 × 10^9^	2.67 × 10^9^	4.73 × 10^7^	1.42 × 10^5^
	rank	3	4	7	8	10	2	6	1	12	11	9	5
*F*_7_	min	2.03 × 10^3^	2.03 × 10^3^	2.09 × 10^3^	2.06 × 10^3^	2.12 × 10^3^	2.04 × 10^3^	2.09 × 10^3^	2.05 × 10^3^	2.18 × 10^3^	2.18 × 10^3^	2.09 × 10^3^	2.07 × 10^3^
	std	3.05 × 10^1^	7.45 × 10^1^	6.12 × 10^1^	3.04 × 10^1^	4.57 × 10^1^	9.70 × 10^1^	3.58 × 10^1^	5.14 × 10^1^	2.20 × 10^1^	7.92 × 10^1^	3.07 × 10^1^	6.91 × 10^1^
	avg	2.09 × 10^3^	2.11 × 10^3^	2.17 × 10^3^	2.11 × 10^3^	2.20 × 10^3^	2.15 × 10^3^	2.15 × 10^3^	2.13 × 10^3^	2.23 × 10^3^	2.28 × 10^3^	2.16 × 10^3^	2.16 × 10^3^
	rank	1	3	9	2	10	6	5	4	11	12	7	8
*F*_8_	min	2.22 × 10^3^	2.23 × 10^3^	2.23 × 10^3^	2.23 × 10^3^	2.24 × 10^3^	2.22 × 10^3^	2.23 × 10^3^	2.22 × 10^3^	2.25 × 10^3^	2.24 × 10^3^	2.24 × 10^3^	2.23 × 10^3^
	std	3.28 × 10^1^	6.72 × 10^1^	1.03 × 10^2^	6.08 × 10^1^	5.04 × 10^1^	4.94 × 10^1^	3.43 × 10^1^	7.42 × 10^1^	1.52 × 10^2^	5.14 × 10^1^	1.11 × 10^2^	5.21 × 10^1^
	avg	2.24 × 10^3^	2.27 × 10^3^	2.31 × 10^3^	2.26 × 10^3^	2.30 × 10^3^	2.26 × 10^3^	2.26 × 10^3^	2.29 × 10^3^	2.54 × 10^3^	2.28 × 10^3^	2.38 × 10^3^	2.26 × 10^3^
	rank	1	6	10	5	9	3	2	8	12	7	11	4
*F*_9_	min	2.48 × 10^3^	2.48 × 10^3^	2.49 × 10^3^	2.48 × 10^3^	2.72 × 10^3^	2.48 × 10^3^	2.49 × 10^3^	2.48 × 10^3^	3.29 × 10^3^	2.79 × 10^3^	2.57 × 10^3^	2.48 × 10^3^
	std	4.93 × 10^−7^	3.29 × 10^−7^	8.43 × 10^1^	1.16 × 10^2^	3.75 × 10^2^	5.08 × 10^−3^	4.18 × 10^1^	2.89 × 10^1^	2.42 × 10^2^	2.61 × 10^2^	5.19 × 10^1^	1.11 × 10^1^
	avg	2.48 × 10^3^	2.48 × 10^3^	2.57 × 10^3^	2.54 × 10^3^	3.36 × 10^3^	2.48 × 10^3^	2.56 × 10^3^	2.50 × 10^3^	3.88 × 10^3^	3.00 × 10^3^	2.65 × 10^3^	2.50 × 10^3^
	rank	2	1	8	6	11	3	7	5	12	10	9	4
*F*_10_	min	2.50 × 10^3^	2.50 × 10^3^	2.50 × 10^3^	2.50 × 10^3^	2.83 × 10^3^	2.50 × 10^3^	2.50 × 10^3^	2.65 × 10^3^	2.59 × 10^3^	2.91 × 10^3^	2.53 × 10^3^	2.70 × 10^3^
	std	1.07 × 10^3^	1.62 × 10^3^	1.51 × 10^3^	1.31 × 10^3^	1.53 × 10^3^	6.97 × 10^2^	8.19 × 10^2^	7.27 × 10^2^	1.26 × 10^3^	1.15 × 10^3^	3.60 × 10^1^	5.59 × 10^2^
	avg	3.10 × 10^3^	4.47 × 10^3^	5.23 × 10^3^	4.34 × 10^3^	5.52 × 10^3^	4.21 × 10^3^	2.85 × 10^3^	4.00 × 10^3^	5.74 × 10^3^	5.91 × 10^3^	2.56 × 10^3^	4.01 × 10^3^
	rank	3	8	9	7	10	6	2	4	11	12	1	5
*F*_11_	min	2.90 × 10^3^	2.90 × 10^3^	3.67 × 10^3^	2.94 × 10^3^	6.17 × 10^3^	2.90 × 10^3^	3.51 × 10^3^	2.90 × 10^3^	7.49 × 10^3^	6.50 × 10^3^	5.58 × 10^3^	2.67 × 10^3^
	std	4.65 × 10^1^	5.04 × 10^1^	1.03 × 10^3^	1.86 × 10^3^	7.77 × 10^2^	4.10 × 10^1^	8.53 × 10^2^	6.31 × 10^1^	7.09 × 10^2^	7.86 × 10^2^	5.57 × 10^2^	1.92 × 10^2^
	avg	2.93 × 10^3^	2.94 × 10^3^	4.65 × 10^3^	4.04 × 10^3^	8.87 × 10^3^	2.92 × 10^3^	4.67 × 10^3^	2.94 × 10^3^	9.22 × 10^3^	8.88 × 10^3^	6.51 × 10^3^	3.06 × 10^3^
	rank	2	3	7	6	10	1	8	4	12	11	9	5
*F*_12_	min	2.93 × 10^3^	2.94 × 10^3^	2.98 × 10^3^	2.95 × 10^3^	3.15 × 10^3^	2.95 × 10^3^	2.95 × 10^3^	2.97 × 10^3^	3.34 × 10^3^	3.01 × 10^3^	3.01 × 10^3^	2.98 × 10^3^
	std	3.73 × 10^1^	4.27 × 10^1^	1.15 × 10^2^	7.72 × 10^1^	1.80 × 10^2^	4.98 × 10^1^	4.13 × 10^1^	5.93 × 10^1^	2.29 × 10^2^	4.45 × 10^2^	6.89 × 10^1^	9.56 × 10^1^
	avg	2.98 × 10^3^	2.99 × 10^3^	3.05 × 10^3^	3.05 × 10^3^	3.58 × 10^3^	3.01 × 10^3^	3.00 × 10^3^	3.06 × 10^3^	3.75 × 10^3^	3.66 × 10^3^	3.13 × 10^3^	3.17 × 10^3^
	rank	1	2	6	5	10	4	3	7	12	11	8	9

**Table 6:** Wilcoxon rank-sum test *p*-values between MDOA and other algorithms for CEC2022 functions.

	DOA	TOC	BKA	COA	SSA	PO	PSO	BWO	GoldSA	DBO	HHO
*F*_1_	2.06 × 10^−6^	1.43 × 10^−7^	6.56 × 10^−3^	6.8 × 10^−8^	1.92 × 10^−7^	9.17 × 10^−8^	4.68 × 10^−2^	6.8 × 10^−8^	6.8 × 10^−8^	6.8 × 10^−8^	1.43 × 10^−7^
*F*_2_	7.11 × 10^−3^	4.54 × 10^−7^	7.95 × 10^−7^	6.8 × 10^−8^	1.67 × 10^−2^	9.17 × 10^−8^	6.56 × 10^−3^	6.8 × 10^−8^	6.8 × 10^−8^	6.8 × 10^−8^	9.75 × 10^−6^
*F*_3_	9.46 × 10^−1^	2.22 × 10^−7^	7.9 × 10^−8^	6.8 × 10^−8^	3.5 × 10^−6^	6.8 × 10^−8^	2.3 × 10^−5^	6.8 × 10^−8^	6.8 × 10^−8^	6.8 × 10^−8^	6.8 × 10^−8^
*F*_4_	6.22 × 10^−4^	2.3 × 10^−5^	4.7 × 10^−3^	6.8 × 10^−8^	5.24 × 10^−5^	1.61 × 10^−4^	9.03 × 10^−1^	6.8 × 10^−8^	6.8 × 10^−8^	6.8 × 10^−8^	6.22 × 10^−4^
*F*_5_	1.33 × 10^−1^	7.71 × 10^−3^	1.58 × 10^−6^	6.8 × 10^−8^	7.9 × 10^−8^	1.23 × 10^−7^	3.64 × 10^−3^	6.8 × 10^−8^	6.8 × 10^−8^	6.8 × 10^−8^	6.8 × 10^−8^
*F*_6_	4.9 × 10^−1^	5.87 × 10^−6^	6.01 × 10^−2^	6.8 × 10^−8^	1.14 × 10^−1^	1.81 × 10^−5^	2.62 × 10^−1^	6.8 × 10^−8^	6.8 × 10^−8^	6.8 × 10^−8^	6.8 × 10^−8^
*F*_7_	1.72 × 10^−1^	4.54 × 10^−6^	9.09 × 10^−2^	1.06 × 10^−7^	1.14 × 10^−2^	8.6 × 10^−6^	1.06 × 10^−2^	6.8 × 10^−8^	6.8 × 10^−8^	1.05 × 10^−6^	1.16 × 10^−4^
*F*_8_	1.29 × 10^−4^	9.75 × 10^−6^	8.36 × 10^−4^	7.58 × 10^−6^	1.06 × 10^−2^	3.29 × 10^−5^	8.59 × 10^−2^	1.23 × 10^−7^	9.75 × 10^−6^	9.13 × 10^−7^	5.9 × 10^−5^
*F*_9_	2.22 × 10^−4^	6.8 × 10^−8^	6.8 × 10^−8^	6.8 × 10^−8^	3.94 × 10^−7^	6.8 × 10^−8^	6.8 × 10^−8^	6.8 × 10^−8^	6.8 × 10^−8^	6.8 × 10^−8^	6.8 × 10^−8^
*F*_10_	6.87 × 10^−4^	1.6 × 10^−5^	6.87 × 10^−4^	3.99 × 10^−6^	1.78 × 10^−3^	5.31 × 10^−2^	1.48 × 10^−3^	2.06 × 10^−6^	7.95 × 10^−7^	1.02 × 10^−1^	1.12 × 10^−3^
*F*_11_	3.64 × 10^−3^	6.8 × 10^−8^	4.54 × 10^−6^	6.8 × 10^−8^	3.85 × 10^−2^	6.8 × 10^−8^	1.23 × 10^−2^	6.8 × 10^−8^	6.8 × 10^−8^	6.8 × 10^−8^	3.64 × 10^−3^
*F*_12_	1.9 × 10^−1^	2.0 × 10^−4^	8.36 × 10^−4^	6.8 × 10^−8^	3.97 × 10^−3^	1.06 × 10^−2^	2.92 × 10^−5^	6.8 × 10^−8^	1.23 × 10^−7^	2.56 × 10^−7^	2.22 × 10^−7^

**Table 7:** Statistical indicators for compression spring problem.

Name	MDOA	DOA	TOC	BKA	COA	SSA	PO	PSO	BWO	GoldSA	DBO	HHO
Best	0.01267	0.01271	0.01267	0.01267	0.01269	0.01267	0.01267	0.01267	0.01642	0.01292	0.01276	0.01268
Std	0.00019	0.00021	0.00152	0.00070	0.00170	0.00203	0.00018	0.00178	181,164,068,739,019	0.00137	0.00139	0.00053
Avg	0.01283	0.01293	0.01335	0.01320	0.01363	0.01379	0.01287	0.01415	137,798,199,953,876	0.01421	0.01357	0.01338

**Table 8:** Wilcoxon rank-sum test results for the compression spring design problem.

DOA	TOC	BKA	COA	SSA	PO	PSO	BWO	GoldSA	DBO	HHO
2.39 × 10^−104^	1.77 × 10^−141^	3.90 × 10^−120^	8.34 × 10^−134^	2.26 × 10^−135^	1.17 × 10^−82^	1.85 × 10^−139^	3.25 × 10^−164^	1.98 × 10^−146^	7.18 × 10^−143^	5.71 × 10^−126^

**Table 9:** Statistical indicators for the minimization of reducer weight.

Name	MDOA	DOA	TOC	BKA	COA	SSA	PO	PSO	BWO	GoldSA	DBO	HHO
Best	2.99 × 10^3^	2.99 × 10^3^	3.01 × 10^3^	3.00 × 10^3^	3.13 × 10^3^	2.99 × 10^3^	3.00 × 10^3^	2.99 × 10^3^	3.90 × 10^11^	3.05 × 10^3^	3.03 × 10^3^	3.01 × 10^3^
Std	1.24 × 10^−8^	1.08 × 10^−4^	6.98 × 10^1^	2.82 × 10^1^	4.26 × 10^13^	9.22 × 10^−5^	2.81 × 10^1^	5.68 × 10^1^	1.41 × 10^14^	5.05 × 10^2^	3.66 × 10^2^	1.83 × 10^1^
Avg	2.99 × 10^3^	2.99 × 10^3^	3.09 × 10^3^	3.02 × 10^3^	2.83 × 10^13^	2.99 × 10^3^	3.03 × 10^3^	3.04 × 10^3^	1.58 × 10^14^	3.31 × 10^3^	3.19 × 10^3^	3.04 × 10^3^

**Table 10:** Wilcoxon rank-sum test results for the reducer weight minimization problem.

DOA	TOC	BKA	COA	SSA	PO	PSO	BWO	GoldSA	DBO	HHO
1.07 × 10^−14^	3.58 × 10^−149^	1.73 × 10^−130^	1.24 × 10^−163^	4.13 × 10^−22^	1.32 × 10^−141^	3.68 × 10^−135^	6.02 × 10^−171^	6.98 × 10^−159^	6.88 × 10^−155^	5.17 × 10^−152^

**Table 11:** Statistical indicators for rolling bearing optimization design problem.

Name	MDOA	DOA	TOC	BKA	COA	SSA	PO	PSO	BWO	GoldSA	DBO	HHO
Best	1.70 × 10^4^	1.70 × 10^4^	1.70 × 10^4^	1.70 × 10^4^	1.70 × 10^4^	1.70 × 10^4^	1.70 × 10^4^	1.70 × 10^4^	3.62 × 10^4^	2.76 × 10^4^	1.71 × 10^4^	1.70 × 10^4^
Std	1.67 × 10^−12^	1.18 × 10^−5^	2.10 × 10^2^	1.47 × 10^3^	5.02 × 10^14^	2.25 × 10^2^	2.81 × 10^2^	4.47 × 10^1^	6.99 × 10^15^	2.04 × 10^3^	7.07 × 10^3^	2.85 × 10^1^
Avg	1.70 × 10^4^	1.70 × 10^4^	1.71 × 10^4^	1.73 × 10^4^	1.12 × 10^14^	1.70 × 10^4^	1.72 × 10^4^	1.70 × 10^4^	3.92 × 10^15^	3.01 × 10^4^	2.66 × 10^4^	1.70 × 10^4^

**Table 12:** Wilcoxon rank-sum test results for the rolling bearing optimization problem.

DOA	TOC	BKA	COA	SSA	PO	PSO	BWO	GoldSA	DBO	HHO
8.51 × 10^−18^	1.05 × 10^−144^	3.47 × 10^−136^	1.14 × 10^−163^	5.97 × 10^−116^	2.46 × 10^−133^	9.61 × 10^−116^	2.20 × 10^−166^	2.28 × 10^−161^	6.01 × 10^−160^	1.67 × 10^−142^

**Table 15:** Comparison results for evaluation indicators.

Model	MAE	MAPE	MSE	RMSE	*R* ^2^
BP	38.8574	11.3980	2352.6741	48.5044	0.8339
DOA-BP	34.8888	9.6561	1974.6982	44.0391	0.8653
TOC-BP	30.3377	8.5143	1517.0216	38.4516	0.8929
BKA-BP	30.6075	8.6478	1518.0896	38.6668	0.8928
COA-BP	31.5702	8.8494	1765.6700	41.3064	0.8753
SSA-BP	30.8464	8.5923	1540.7173	38.7951	0.8912
PSO-BP	30.2390	8.3596	1467.3263	37.5683	0.8964
BWO-BP	31.5669	8.8754	1534.1592	38.8394	0.8917
GoldSA-BP	33.3740	9.2713	1880.1424	42.6126	0.8673
DBO-BP	34.0081	9.7200	1836.5508	42.3941	0.8703
HHO-BP	29.9543	8.2290	1440.3682	37.5512	0.8983
MDOA-BP	28.1804	7.8631	1231.8375	35.0104	0.9130

**Table 13:** Statistical indicators for tubular column design problem.

Name	MDOA	DOA	TOC	BKA	COA	SSA	PO	PSO	BWO	GoldSA	DBO	HHO
Best	26.48599	26.48599	26.48599	26.48599	26.48609	26.48599	26.48706	26.48599	26.54314	26.50160	26.48975	26.48633
Std	1.09 × 10^−14^	1.01 × 10^−9^	1.27 × 10^−1^	2.51 × 10^−4^	3.07 × 10^−2^	1.53 × 10^−7^	2.19 × 10^−2^	1.09 × 10^−14^	1.29 × 10^−1^	2.15 × 10^−1^	1.68 × 10^−1^	3.11 × 10^−2^
Avg	26.48599	26.48599	26.51456	26.48609	26.50752	26.48599	26.50672	26.48599	26.80228	26.71989	26.60771	26.52431

**Table 14:** Wilcoxon rank-sum test results for the tubular column design problem.

DOA	TOC	BKA	COA	SSA	PO	PSO	BWO	GoldSA	DBO	HHO
8.51 × 10^−18^	1.05 × 10^−144^	3.47 × 10^−136^	1.14 × 10^−163^	5.97 × 10^−116^	2.46 × 10^−133^	9.61 × 10^−116^	2.20 × 10^−166^	2.28 × 10^−161^	6.01 × 10^−160^	1.67 × 10^−142^

## Data Availability

The original contributions presented in this study are included in the article. Further inquiries can be directed to the corresponding author.
